# Uptake of newer methodological developments and the deployment of meta-analysis in diagnostic test research: a systematic review

**DOI:** 10.1186/1471-2288-11-27

**Published:** 2011-03-14

**Authors:** Brian H Willis, Muireann Quigley

**Affiliations:** 1 Biostatistics, University of Manchester, Jean McFarlane building, Oxford Road, Manchester, M13 9PL, UK; 2 Centre for Social Ethics and Policy, University of Manchester, Williamson building, Oxford Road, Manchester, M13 9PL, UK

## Abstract

**Background:**

The last decade has seen a number of methodological developments in meta-analysis of diagnostic test studies. However, it is unclear whether such developments have permeated the wider research community and on which applications they are being deployed. The objective was to assess the uptake and deployment of the main methodological developments in the meta-analysis of diagnostic tests, and identify the tests and target disorders most commonly evaluated by meta-analysis.

**Methods:**

*Design *- systematic review. *Data Sources *- Medline, EMBASE, CINAHL, Cochrane, PsychInfo, Global health, HMIC, and AMED were searched for studies published before 31^st ^December 2008. *Selection criteria *- studies were included if they satisfied all of the following: evaluated a diagnostic test; measured test performance; searched two or more databases; stated search terms and inclusion criteria; used a statistical method to summarise performance. *Data extraction *- included the following data items: year; test; reference standard; target disorder; setting; statistical and quality methods.

**Results:**

236 studies were included. Over the last 5 years the number of meta-analyses published has increased, but the uptake of new statistical methods lags behind. Pooling the sensitivity and specificity and using the SROC remain the preferred methods for analysis in 70% of studies, with the bivariate random effects and HSROC model being used in only 22% and 5% of studies respectively. In contrast, between 2006 and 2008 the QUADAS tool was used in 40% of studies. Broadly, radiological imaging was the most frequent category of tests analysed (36%), with cancer (22%) and infection (21%) being the most common categories of target disorder. Nearly 80% of tests analysed were those normally used in specialist settings.

**Conclusion:**

Although quality assessment in meta-analyses has improved with the introduction of QUADAS, uptake of the newer statistical methods is still lagging behind. Furthermore, the focus of secondary research seems to be in evaluating specialist tests in specialist settings, in contrast to the more routine tests and settings encountered in the majority of clinical practice.

## Background

The application of meta-analysis to diagnostic tests has lagged behind its application to therapeutic trials [1,2], emerging only in the last fifteen years. In part, this is due to therapeutic trials benefiting from certain design features not afforded diagnostic studies: the randomised controlled trial design, combined with the use of hard outcomes such as mortality, greatly facilitates the validity of the measured effects reported [1]. Also these measured effects can be adequately summarised by a single statistic such as an odds ratio [3].

In contrast, test accuracy studies tend to be cross sectional in design [4,5], in order to adequately reflect the influence that the patient case-mix or 'spectrum' may have on a test's performance [6,7]. This is measured in terms of the test's ability to discriminate those with disease from those without. Unfortunately, obtaining a subject's true disease status is not always free from ambiguity and relies upon the fidelity of the reference standard [8]. Even with satisfactory study design and ascertainment of disease status, measuring performance is hampered by there not being a single adequate statistic [9] which both describes its accuracy and has clinical utility in practice.

Hence two statistics, the sensitivity and specificity, are normally used when summarising a test's performance, and via Bayes' theorem [10] these have the added benefit of being useful to practitioners. The complication is that they are not independent: both vary with the threshold for a positive test result [11]. While this has long been recognised, and may be graphically depicted by a receiver operator characteristic (ROC) curve [12,13], it is only recently that the association has been incorporated within meta-analysis.

Prior to this, the main method for combining studies was to pool performance characteristics separately using the same fixed [14] and random effects [15] models developed in therapeutics. Early attempts at including the association between the sensitivity and specificity involved making the assumption that the only source of variation between studies (heterogeneity) was due to changes in the test threshold. This is the basis of the summary receiver operating characteristic (SROC) curve [16,17].

The potential to include other sources of heterogeneity in models has existed for some time. Yet, it is only in the last decade that two other approaches, the bivariate random effects model (BRM)[18,19] and hierarchical SROC (HSROC) model [20], have emerged that place between-study variation of the sensitivity and specificity on a firm statistical basis.

Developments in statistical methodology have been mirrored by advances in methods for assessing the quality of studies for systematic reviews. This led to the publication of the Quality Assessment of Diagnostic Accuracy Studies (QUADAS) tool in 2004 [21]. The Standards for the Reporting of Diagnostic accuracy studies (STARD) initiative [5] gave a reporting framework for investigators that undoubtedly improved the quality of primary studies. Nonetheless, quality assessment had lacked a standardised approach with a number of general and ad hoc tools being suggested over the last twenty years [22-26]. As a 14 point questionnaire the QUADAS tool [21] provides investigators with a means of assessing the major domains that affect a diagnostic study's validity. If applied consistently, this may facilitate inter-study comparisons.

Thus, over the last decade, we have seen significant progress in the methodology used in the meta-analysis of diagnostic tests. Unfortunately, some of this progress has been at the cost of increased complexity that may inhibit its implementation. It is of interest to establish whether such developments have permeated the wider research community. The objective of this systematic review was to assess the uptake and deployment of the main methodological developments in the meta-analysis of diagnostic tests, and to identify the diagnostic tests and target disorders most commonly evaluated by meta-analysis.

## Methods

### Search Strategy

A search strategy was developed aimed at retrieving articles in secondary research. The following databases were searched using an OVID interface: Medline, EMBASE, CINAHL, PsychInfo, Global Health, Cochrane, HMIC and AMED. The initial search strategy was conducted in October 2008 and again in September 2009 to identify studies published before the 31^st ^December 2008. The search strategies for each of the databases are in Additional file 1. All searches and the removal of duplicate citations were carried out by one of the reviewers.

### Inclusion Criteria

For the purpose of the review, a meta-analysis was considered to be a special type of systematic review in which the accepted methods used to construct a systematic review were followed and the results were aggregated by statistical methods. A number of other terms were also defined to remove ambiguity in the inclusion criteria and to aid reproducibility; the full details of these may be found in Additional file 2. They were developed as an algorithm with the first criterion being applied to the titles and abstracts of all the citations. Full text articles were then retrieved on those which either satisfied the first criterion, or when there was insufficient information to make a decision.

Studies were included if they satisfied all of the following criteria: the citation was an original study of a diagnostic or screening test; one of the objectives was to estimate an accepted performance measure of the test; two or more of the major electronic databases were searched; three or more search terms were explicitly stated in the methods; at least one statistical method was used to combine the studies and summarise the performance of the test.

### Data Extraction and appraisal

Data were abstracted on the following: year; test; reference standard; target disorder; patient setting; statistical methods and correlation; presence of heterogeneity; and quality assessment tool. Four statistical methods were specifically searched for as part of the appraisal: pooling of performance characteristics using a fixed effects [14] or random effects [15] model; SROC method as described by Moses and colleagues [16,17]; bivariate random effects model (BRM) [18,19]; the HSROC curve as proposed by Rutter and Gatsonis [20]. Similarly, use of the QUADAS tool [21] was specifically noted.

The selection, data extraction and the appraisal of studies were conducted by a single reviewer (BHW). A second reviewer (MQ) independently carried out the selection process on a 10% random sample drawn from the unduplicated set of citations and also independently extracted and appraised a 10% random sample of the included studies. The Kappa statistic was used to estimate the level of agreement [27,28] interpretation was based on recognised criteria [28,29].

### Statistical analysis

In addition to inspection, the frequency distributions of the different methods used were compared. The baseline measures for comparison were: the number of studies applying a particular statistical method per year; and the proportion of the number included studies published per year reporting the method of interest. The respective frequency distributions were compared using the non-parametric Kolmogorov-Smirnov statistic (D) [30] and the Wilcoxon signed rank test statistic (V) [31]. These are appropriate when little is known about the distributions being evaluated. As the BRM and the HSROC model have been shown, in most cases to be statistically equivalent [32,33], their respective distributions were combined when comparing them with the other methods. A value of p < 0.05 was considered significant. The broad categories of tests, target disorders and patient settings evaluated in the included studies were summarised using pie charts.

## Results

### Citation yield

The searches produced 6259 citations, which after removal of duplicate citations were reduced to 4336 studies. Screening of the titles and abstracts using the first criterion excluded a further 3288 studies (see Additional file 3 for flowchart). Of the remaining studies, the selection criteria were applied to the full text manuscripts; in nine studies the complete manuscripts were not retrieved. Full text review excluded a further 803 studies, leaving 236 studies to be included for appraisal (see Additional file 4). For the selection process the Kappa score was 0.86 indicating 'near perfect agreement' between the two reviewers [29].

### Uptake and implementation of methods

**Figure 1:**
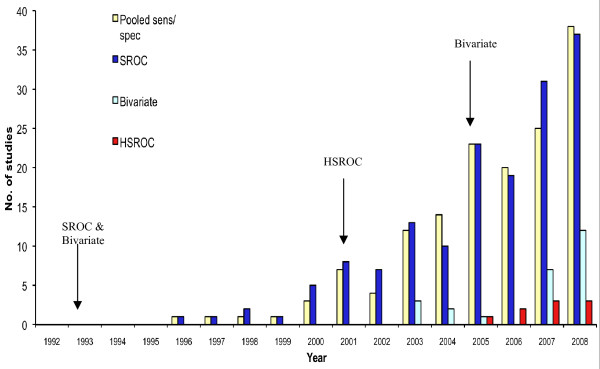
**The distribution of included studies per statistical method per year**. Also shown is the year of publication of the seminal papers corresponding to the statistical methods (arrows).

From figure 1 it is clear that the number of studies, which met the inclusion criteria has increased markedly over the last decade. It also shows the distribution of statistical methods used.

Part of the impetus behind developing more advanced methods [18-20] was the recognition of widespread heterogeneity in diagnostic studies. Accordingly, this was reported in 70% of the included studies. Despite their limitations, overall the most popular methods remain the SROC curve, which was applied in 158 of the studies, and independent pooling of the sensitivity and the specificity, which was used in 151 studies. In 107 studies, both methods were used and there were no significant differences between these two distributions (V = 9, p = 0.11; and D = 0.36, p = 0.33).

A simple approach to modelling heterogeneity is that deployed in the SROC curve, where the transformed sensitivity and specificity are assumed to follow a linear relationship [16,17]: an assumption, which may be tested by calculating the correlation coefficient [34,35]. Despite this, the correlation coefficient was reported in only 24 (15%) of the studies, which used an SROC curve model.

In contrast to the two previous methods, the uptake of the BRM and HSROC model since their introduction has been limited: twenty-five studies used a BRM and only nine used an HSROC model. Unsurprisingly, their combined distribution was significantly different from that of independent pooling (V = 21, p = 0.03; and D = 1, p = 0.002) and the SROC curve (V = 21, p = 0.03; and D = 1, p = 0.002). The year of publication of the seminal papers relating to each of these methods is also shown. It is of interest that the BRM was probably first proposed in 1993 [18], the same year the SROC was proposed [16,17]. This study was referenced by five of the meta-analyses. However, in the field of diagnostics, the most quoted reference for the BRM is that of Reitsma and colleagues in 2005 [19], which was cited in 18 studies.

An interesting question is whether using the new methods (BRM/HSROC) significantly alters the summary statistics from those derived using the more traditional methods (independent pooling/SROC). A number of studies reported using more than one statistical method to summarise the data. Yet, only one study explicitly compared the summary performance statistics derived from independent pooling with those from an HSROC curve model [36]. In this study, the summary statistics were not significantly different [36].

**Figure 2:**
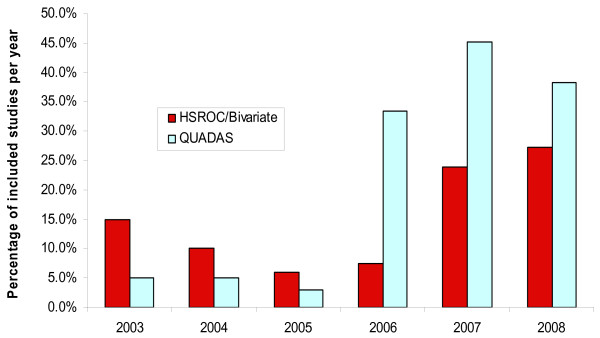
**Uptake of the new statistical methods (bivariate and HSROC) compared with the uptake of the quality assessment tool QUADAS**.

Having been recently introduced, and presenting fewer technical barriers to its implementation, the uptake of the QUADAS tool by researchers provides a useful comparison with that of the new statistical methods (figure 2). Although 69 studies cited its use, only 51 used it in either its entirety or a substantial part of it in assessing quality. Overall, statistical comparisons showed no significant differences between the distributions in figure 2, (V = 15, p = 0.44; and D = 0.5, p = 0.44). However, the recent trend (last 3 years) is that the use of QUADAS exceeded the combined use of the BRM and the HSROC model by over 10% each year.

An error that is sometimes made when implementing quality assessment tools is to sum the scores of the individual categories. Total scores are based on the implicit assumption that the different domains on quality are comparable on a linear scale, which has no validity. Yet, nearly half (49%) of the studies using the QUADAS tool reported an overall quality scores.

The evidence tables for the individual studies may be found in Additional file 5.

### 2. Characteristics of the included studies

**Figure 3:**
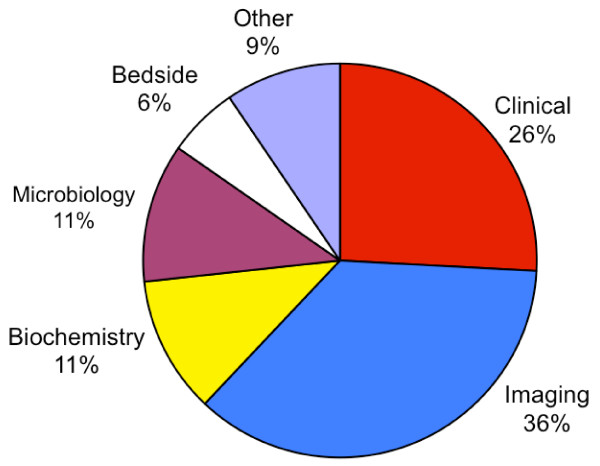
**Distribution of broad categories of tests analysed**.

**Figure 4:**
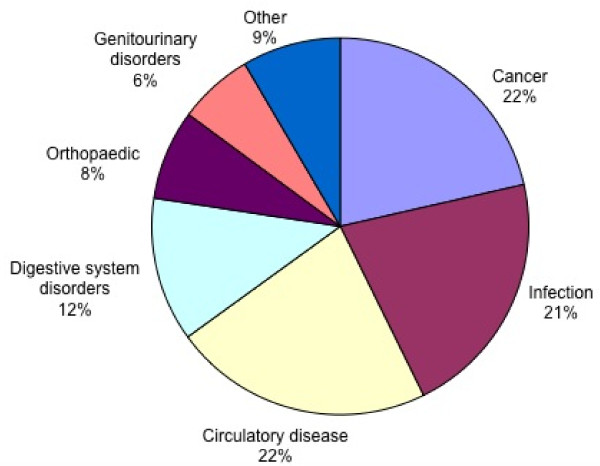
**Distribution of tests analysed per target disorder category**.

**Figure 5:**
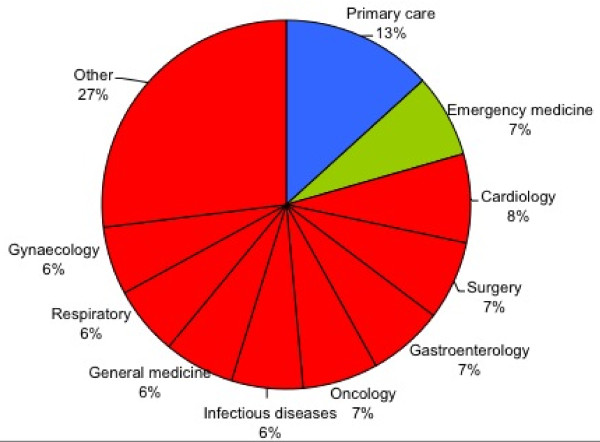
**Distribution of tests analysed per patient setting**. Secondary care specialties shown in red.

The included studies frequently evaluated more than one test, on more than one target disorder, and in more than one setting. There were a total of 685 separate test evaluations, which were analysed in terms of category of test (figure 3), category of target disorder (figure 4) and patient setting (figure 5). Classification was based on a combination of evidence from the individual studies, guidance from ICD-10 [37] and clinical experience. Note that in the following paragraphs, numbers given represent the number of separate evaluations, not the number of studies.

Radiological imaging tests were the most common type of test appraised, with ultrasound (69), magnetic resonance (64) and computer tomography (53) being the most frequent technologies evaluated (figure 3). Although 26% of tests analysed were categorised as clinical, this total is somewhat skewed by six studies, which accounted for nearly half of the clinical tests (figure 3).

Broadly, cancer (147) and infection (145) were the most frequent categories of target disorder appearing in the included studies (figure 4) with tuberculosis (50), stable coronary artery disease (36) and gallstones and cholecystitis (33) being the most common conditions. Although the investigation of cancer varied, a common theme was the evaluation of imaging technologies to stage the disease after the primary lesion had already been identified. Ischaemic heart disease (87) also appeared repeatedly as a target disorder of interest, either as an acute manifestation (acute coronary syndrome, or myocardial infarction) or as a chronic one (coronary artery disease, exertional angina).

The typical populations in which the tests would be applied in practice are shown in figure 5. The most striking feature from the tests evaluated is the proportion that would be used in a secondary (or greater) care setting. Although there is an even distribution across the sub-specialties, 87% of the diagnostic tests in the studies are hospital based (figure 5).

A description of the characteristics for the individual studies may be found in Additional file 6.

## Discussion

### Summary of evidence

Over the past decade the number of meta-analyses published in diagnostic research has clearly increased. Although our stricter criteria excluded some of the earlier publications, the recent surge in reporting is both a reflection of increased interest in the field and greater attention to quality by investigators.

Notwithstanding the obvious interest and likely improvement in standards, meta-analysis in diagnostic research still poses a number of difficulties to researchers. The studies appraised in this review seemed to demonstrate a lack of consensus on how best to aggregate results: in nearly two thirds of studies two or more statistical methods were used and in some instances up to four methods were employed.

The SROC curve [16,17] and pooling of the sensitivity and specificity [14,15] remain the most popular methods for summarising diagnostic studies, despite both having technical shortcomings. To overcome these deficiencies the bivariate random effects [18,19] and the HSROC model [20] were independently proposed; but their use by research groups in the studies appraised was limited.

As part of the normal process a delay between proposal and deployment by the wider research community should be expected. If the uptake of the SROC curve is representative, then figure 1 shows that it could be at least 10 years before researchers substantially apply the new models. However, this does not explain the discrepancy between the use of the new statistical methods and the QUADAS tool [21], which in the last three years was more widely applied by investigators (figure 2).

Whereas pooling the sensitivity and the specificity or constructing an SROC curve may be easily achieved using, for example, a spreadsheet, the same cannot be said of either of the newer models. Both of these use maximum likelihood estimation of the population parameters and in the case of the HSROC model, Monte Carlo simulation may be required, particularly when a Bayesian approach is taken [20,38]. Thus, specialist statistical software is needed and with it a technical knowledge [9] which may not be available to a number of research groups.

To widen access, software packages have recently become available which incorporate the BRM and HSROC models. These include: METANDI written in STATA [39]; METADAS written in SAS [39]; and DiagMeta [40] and INLA [41] both written in R. The latter two, being written in R, have the advantage of being freely available on the Internet. However, even with increasing availability of dedicated software, for those without a technical knowledge they remain 'black boxes'. This may, in part, explain a lack of uptake of the new methods.

Perhaps an equally important issue is the relevance of the results produced by meta-analysis. The SROC curve model gives a performance curve with no indication of the corresponding threshold for individual points on the curve. The two newer models are able to produce summary estimates of the sensitivity and specificity. These take into account all possible variances within and between the studies analysed, yet do not say how a test may perform in any particular setting [9]. Recently there has been an argument in favour of moving towards prediction regions, which define the region where the true performance of future studies conducted may lie [9,32]. However, this just provides a further iteration to the research process without necessarily addressing the issue of transferability of results into practice.

There has undoubtedly been a shift in the thinking on meta-analysis towards a multivariate approach, as it is considered more 'statistically rigorous' than other methods [9].

Statistical rigour in itself may not be a sufficient argument for advocating uptake of the new bivariate methods; particularly, if the summary estimates generated are not significantly different from those derived from simpler univariate methods. There were no significant differences in the one study identified in this review that compared the two approaches [36]. However, Harbord and colleagues have found that estimates of the sensitivity and specificity may differ by greater than 10% in some cases [9]. Such differences do not necessarily translate into changes in clinical decisions: Simel and Bossuyt found only modest differences in estimates of the likelihood ratios and importantly, posterior probabilities, when univariate and bivariate methods were compared [42].

It seems this new paradigm is going through an articulation process where its boundaries and uses are still being defined [9,43,44]. One potential use for the new methods is in comparative analyses between two different tests [3], in order to determine which test is more accurate overall without knowing their individual accuracies. But as they currently stand the transferability of summary estimates from the new methods is far from clear.

The distribution of tests analysed in the included studies is also telling, demonstrating incongruence between research and patient need. The diagnostic process has been notably analysed in the early work of Crombie, Hampton *et al*, and later Sandler [45-47]. These showed that between 70-90% of the patients are correctly diagnosed clinically by the history and examination [45-47]. Yet, in the secondary research analysed here, clinical tests represented only 26% of the diagnostic tests analysed. This imbalance is unlikely to change as priority is given to the evaluation of emerging new technologies [48,49].

There is similar incongruence between the settings where most patients are seen in practice and the settings where the tests were analysed. The vast majority of patients in practice are either seen in a primary care or an emergency care setting [50,51], but it seems that secondary research, at least, is being directed towards evaluating diagnostic tests which are mainly used in specialist settings.

Designing a study to evaluate a clinical test particularly in a primary care setting is hampered by the availability of an adequate reference standard [52]. The reference standards which involve imaging, histology, or a specialist diagnosis are more likely to be available in a secondary care setting than a primary care setting.

Also applying a suitable reference standard to primary care patients may be constrained by ethical considerations; particularly, if the patient population is considered low risk, but the reference standard carries potential hazards. This is not the case when the test is being used to stage an already identified cancer. Here there is a significant risk of inappropriate treatment and decreased life expectancy by not performing such a test for the patient. This, in part, explains the number of studies evaluating imaging technologies to stage patients with cancer.

### Limitations of study

This study has some limitations. A single reviewer performed both the study selection and the data extraction. To measure the reproducibility, a second reviewer, blinded to the results, independently carried out each of these processes on two separate 10% random samples. Agreement between the reviewers was measured using the kappa statistic and this demonstrated 'good to excellent agreement' for both the study selection and data extraction. Nonetheless, this method is still more likely to yield errors over the preferred method of complete, independent replication of both steps by at least two reviewers.

What may be considered a meta-analysis of diagnostic test studies is not without ambiguity. The definition used here was one, which encompassed sound systematic review principles and used recognised methods to aggregate the results. Inevitably such a definition leads to tight inclusion criteria and this is reflected by the number of studies included, which are perhaps fewer than might be expected from earlier reviews of the field [53].

## Conclusions

In summary, there is still limited uptake of the new statistical methods used in meta-analysis of diagnostic test studies. Although this should change with increased availability of statistical software, it remains unclear to what extent these methods will actually benefit clinical practice.

More broadly, if the studies reviewed here are representative of diagnostic research in general, there appears to be a disconnect between the focus of investigators on specialist tests in specialist settings and the type of tests and settings actually encountered in the majority of clinical practice.

## Competing interests

The authors declare that they have no competing interests.

## Authors' contributions

Both BHW and MQ selected, extracted and appraised the data. BHW wrote the first draft. MQ commented on all drafts. All authors read and approved the final manuscript.

## Pre-publication history

The pre-publication history for this paper can be accessed here:

http://www.biomedcentral.com/1471-2288/11/27/prepub

## Supplementary Material

Additional file 1 **Appendix 1**. search algorithms. Click here for file

Additional file 2 **Appendix 2**. Detailed description of inclusion criteria. Click here for file

Additional file 3 **Appendix 3**. Flowchart showing inclusion/exclusion decisions. Click here for file

Additional file 4 **Appendix 4**. Set of included studies. Click here for file

Additional file 5 **Appendix 5**. Evidence tables for individual studies. Click here for file

Additional file 6 **Appendix 6**. Description of included studies. Click here for file
