# Supplementary material for: Uptake of newer methodological developments and the deployment of meta-analysis in diagnostic test research: a systematic review
Source: BMC Med Res Methodol. 2011 Mar 14;11:27. doi: 10.1186/1471-2288-11-27 (PMC3065444; doi:10.1186/1471-2288-11-27)
Supplement: Additional file 1 — Appendix 1. search algorithms. [file 1471-2288-11-27-S1.DOC]

**Appendix 1**: **Search algorithms**

|  | **MEDLINE SEARCH** |  |  | **EMBASE SEARCH** |
| --- | --- | --- | --- | --- |
| 1 | exp diagnosis/ |  | 1 | exp "sensitivity and specificity"/ |
| 2 | diagnos$.mp. |  | 2 | (odds adj ratio$).mp. |
| 3 | exp Mass Screening/ |  | 3 | exp Area Under the Curve/ |
| 4 | exp "Sensitivity and Specificity"/ |  | 4 | (sensitivit$ and specificit$).mp. |
| 5 | exp odds ratio/ |  | 5 | exp RECEIVER OPERATING CHARACTERISTIC/ |
| 6 | exp Area Under Curve/ |  | 6 | (receiver adj operat$).mp. |
| 7 | (sensitivit$ and Specificit$).mp. |  | 7 | (likelihood adj ratio$).mp. |
| 8 | (likelihood adj ratio$).mp. |  | 8 | exp meta analysis/ |
| 9 | ROC$1.mp. |  | 9 | meta-analys$.mp. |
| 10 | (receiver adj operat$).mp. |  | 10 | meta-regression.mp. |
| 11 | 1 or 3 or 2 |  | 11 | exp DIAGNOSIS/ |
| 12 | 8 or 6 or 4 or 7 or 10 or 9 or 5 |  | 12 | exp SCREENING/ |
| 13 | 11 and 12 |  | 13 | diagnos$.mp. |
| 14 | exp Meta-Analysis/ |  | 14 | 6 or 4 or 1 or 3 or 7 or 2 or 5 |
| 15 | exp meta-analysis as topic/ |  | 15 | 8 or 10 or 9 |
| 16 | meta-analys$.mp. |  | 16 | 11 or 13 or 12 |
| 17 | meta-regression.mp. |  | 17 | 16 and 15 and 14 |
| 18 | 16 or 17 or 15 or 14 |  |  |  |
| 19 | 18 and 13 |  |  |  |
