# Supplementary material for: Uptake of newer methodological developments and the deployment of meta-analysis in diagnostic test research: a systematic review
Source: BMC Med Res Methodol. 2011 Mar 14;11:27. doi: 10.1186/1471-2288-11-27 (PMC3065444; doi:10.1186/1471-2288-11-27)
Supplement: Additional file 2 — Appendix 2. Detailed description of inclusion criteria. [file 1471-2288-11-27-S2.DOC]

**Appendix 2**: **Inclusion criteria**.

For the purpose of this review a meta-analysis was considered to be a special type of systematic review in which the accepted methods used to construct a systematic review were followed and the results were aggregated by statistical methods.

There were a number of considerations when designing these inclusion criteria. The first was to try and make the criteria unambiguous by defining terms which are otherwise open to interpretation such as ‘original’, ‘diagnostic study’, and ‘acceptable outcomes’. The second was to make the criteria explicit and as part of a step-by-step algorithm to facilitate reproducibility. The third was to ensure the criteria captured the desired studies. In particular the meta-analyses should answer an objective question; the investigators should have ensured that searches were as complete as possible; reporting should be sufficient to allow others to reproduce the work; and accurate and acceptable quantitative inferences were made from the results.

As previous work has shown the potential to miss valuable studies when the searches are confined to a single database [51, 52], only those meta-analyses, which had resulted from the searching of at least 2 electronic databases were included. Furthermore included studies were restricted to the English language.

The inclusion criteria are stated more explicitly below.

***Part A*** *From reading the title and abstract*

**1. Is the citation an original study of a diagnostic/screening test?**

There were three parts to this:

a. *Original* is defined as a primary or secondary (systematic review) evaluation of the technology. eg editorials, commentaries and narrative reviews are excluded at this stage.

b. *A diagnostic/screening test* is defined as a technology aimed at identifying a target disorder which is present at the time of testing.

The target disorder is a pathological process and not related to a success or failure of an intervention. Thus a test, which ascertains the correct placement of a stent, is not a diagnostic test, since the target is merely whether the device was placed correctly. However, a test, which evaluates the degree of stenosis of a stent following successful placement would be considered a diagnostic test, as the stenosing of a stent is a pathological process.

c. Technologies, which predict the future occurrence of a target disorder, which is not present at the time of testing, are excluded at this stage.

***Part B*** *To identify the systematic reviews - from reading the full citation*

**2.** **Is one of the objectives of the study to estimate a measure of the performance of the diagnostic test?**

Acceptable performance measures are:-

a. Estimates of the sensitivity and specificity as a pair.

b. Diagnostic odds ratio (=LR+/LR-) only.

c. Likelihood ratios

d. A ROC curve or other model for combining the above

Other measures such as non-diagnostic odds ratio’s, relative risk, predictive values are not sufficient to be included.

Where point estimates are given then associated confidence intervals should be given. Where the study has multiple objectives one of the objectives must satisfy the above to be included.

**3. Did the investigators of the study search two or more of the major databases to identify the relevant articles?**

Examples include – Medline, Embase, CINAHL, HealthStar, EconLit

**4. Did the investigators of the study explicitly state the search terms in the methods?**

Preferably search algorithms in the appendices should be stated, but minimally 3 or more search terms including MESH headings

**5. Were the inclusion criteria explicitly stated in the methods?**

***Part C*** *Identifying the Meta-analyses*

**6. Did the investigators use at least one statistical method to quantitatively combine the studies and summarise the performance of the test?**

If pooling of performance characteristics (such as sensitivity or specificity) was all that was done then minimally confidence intervals should be calculated

References

[44] Golder S, McIntosh HM, Duffy S, Glanville J, Centre for Reviews and Dissemination and UK Cochrane Centre Search Filters Design Group. Developing efficient search strategies to identify reports of adverse effects in MEDLINE and EMBASE. Health Information and Libraries Journal 2006; 23: 3-12

[45] Whiting P, Westwood M, Burke M, Sterne J, Glanville J Systematic reviews of test accuracy should search a range of databases to identify primary studies Journal of Clinical Epidemiology 61 (2008) 357-364
