# Supplementary material for: Uptake of newer methodological developments and the deployment of meta-analysis in diagnostic test research: a systematic review
Source: BMC Med Res Methodol. 2011 Mar 14;11:27. doi: 10.1186/1471-2288-11-27 (PMC3065444; doi:10.1186/1471-2288-11-27)
Supplement: Additional file 3 — Appendix 3. Flowchart showing inclusion/exclusion decisions. [file 1471-2288-11-27-S3.DOC]

**Appendix 3**

Flowchart of studies showing results of applying the inclusion criteria.

Also shown are the types of study or reasons for exclusion

Unduplicated citations retrieved from searches

Apply step 1 to title and abstract

Retrieve full text articles

Step 1 full text review

Step 2

Include 1048

Outstanding 9

Step 6

Included 236

4336

Exclude 3288

Narrative reviews, editorials, abstracts & foreign language studies. Eight were publications also published as HTA reports

Unable to locate 9

Retrieved 1039

Step 3

Step 4

Step 5

Include 881

Include 751

Include 384

Include 328

Include 304

Exclude 158

Exclude 130

Exclude 367

Exclude 56

Exclude 24

Exclude 68

Abstracts, editorials & narrative reviews, or not a diagnostic studies

Did not ascertain acceptable diagnostic measures

Did not list 3 or more search terms

Did not search 2 or more electronic databases

Did not explicitly state inclusion criteria

Did not use acceptable summary statistical methods.
