# Supplementary material for: Uptake of newer methodological developments and the deployment of meta-analysis in diagnostic test research: a systematic review
Source: BMC Med Res Methodol. 2011 Mar 14;11:27. doi: 10.1186/1471-2288-11-27 (PMC3065444; doi:10.1186/1471-2288-11-27)
Supplement: Additional file 5 — Appendix 5. Evidence tables for individual studies. [file 1471-2288-11-27-S5.DOC]

**Appendix 5 - Evidence tables for individual studies**

| **Reference** | **Year** | **Pooled sen/spe** | **Pooled LR** | **Pooled DOR** | **SROC** | **Bivariate** | **HSROC** | **Other** | **Techniques per paper** | **Hetero- geneity** | **Correl- ation** | **Quadas** | **Quadas Score** |
| --- | --- | --- | --- | --- | --- | --- | --- | --- | --- | --- | --- | --- | --- |
| Abdulla | 2007 | yes | yes | yes | yes |  |  |  | 4 | yes |  |  |  |
| Abubakar | 2007 | yes |  | yes | yes |  |  |  | 3 | yes |  | Total |  |
| Akcil | 2008 | yes |  |  | yes |  |  |  | 2 | yes |  |  |  |
| Arbyn | 2008 |  |  |  | yes |  | yes |  | 2 | yes |  |  |  |
| Arbyn | 2004 | yes |  |  |  |  |  |  | 1 | no |  |  |  |
| Atieh | 2008 | yes | yes | yes | yes |  |  |  | 4 | yes |  |  |  |
| Bafounta | 2001 |  |  |  | yes |  |  |  | 1 | Unclear |  |  |  |
| Bafounta | 2004 |  |  |  | yes |  |  |  | 1 | Unclear |  |  |  |
| Bagai | 2006 |  | yes |  |  |  |  |  | 1 | Unclear |  |  |  |
| Bakis | 2004 | yes |  |  | yes |  |  |  | 2 | no |  |  |  |
| Barnes | 2002 | yes |  |  | yes |  |  |  | 2 | yes |  |  |  |
| Bastian | 1998 | yes |  |  |  |  |  |  | 1 | Unclear |  |  |  |
| Benjaminse | 2006 | yes | yes | yes |  |  |  |  | 3 | Unclear |  |  |  |
| Berner | 2007 | yes | yes | yes | yes |  |  |  | 4 | yes |  | Subs | yes |
| Berry | 1999 |  |  |  | yes |  |  |  | 1 | Unclear |  |  |  |
| Berry | 2002 |  |  |  | yes |  |  |  | 1 | yes |  |  |  |
| Bipat | 2004 |  |  |  | yes | yes |  |  | 2 | yes |  |  |  |
| Bipat | 2003 |  |  |  |  | yes |  |  | 1 | yes |  |  |  |
| Bipat | 2005 |  |  |  |  | yes |  |  | 1 | Unclear |  |  |  |
| Blacksell | 2006 | yes | yes | yes | yes |  |  |  | 4 | yes |  | Total | yes |
| Brealey | 2005 | yes |  | yes | yes |  |  |  | 3 | yes |  |  |  |
| Brown | 2003 | yes |  | yes | yes |  |  |  | 3 | yes |  |  |  |
| Brown | 2002 | yes |  |  | yes |  |  |  | 2 | yes |  |  |  |
| Bruyninckx | 2008 | yes | yes | yes |  |  |  |  | 3 | yes |  | Total |  |
| Burr | 2007 |  |  | yes | yes |  | yes |  | 3 | Unclear |  | Total |  |
| Campens | 1997 | yes |  |  |  |  |  |  | 1 | yes |  |  |  |
| Castilla-Rilo | 2007 | yes |  |  | yes |  |  |  | 2 | Unclear |  |  |  |
| Cavallazzi | 2008 |  |  | yes | yes |  |  |  | 2 | no |  |  |  |
| Cepoiu | 2008 | yes |  |  | yes |  |  |  | 2 | yes |  |  |  |
| Chalco | 2005 | yes | yes | yes |  |  |  |  | 3 | yes |  |  |  |
| Chen | 2001 |  |  |  | yes |  |  |  | 1 | yes |  |  |  |
| Chua | 2008 | yes |  |  | yes |  |  |  | 2 | no |  | Total | yes |
| Clark | 2001 |  | yes |  |  |  |  |  | 1 | yes |  |  |  |
| Clark | 2002 | yes | yes |  |  |  |  |  | 2 | yes |  |  |  |
| Clark | 2000 | yes |  |  |  |  |  | yes | 2 | yes |  |  |  |
| Colin | 2001 | yes |  |  |  |  |  |  | 1 | yes |  |  |  |
| **Reference** | **Year** | **Pooled sen/spe** | **Pooled LR** | **Pooled DOR** | **SROC** | **Bivariate** | **HSROC** | **Other** | **Techniques per paper** | **Hetero- geneity** | **Correl- ation** | **Quadas** | **Quadas Score** |
| Dales | 1990 | yes |  |  |  |  |  |  | 1 | yes |  |  |  |
| de Bondt | 2007 |  |  |  | yes | yes |  |  | 2 | yes |  |  |  |
| de Kroon | 2003 | yes | yes |  | yes |  |  |  | 3 | yes | yes |  |  |
| Debrey | 2008 | yes |  |  | yes |  |  |  | 2 | yes |  | Partial |  |
| Delgado-Bolton | 2003 | yes | yes |  | yes |  |  |  | 3 | no |  |  |  |
| Deville | 2000 |  |  | yes | yes |  |  |  | 2 | yes |  |  |  |
| Deville | 2004 | yes |  | yes | yes |  |  |  | 3 | yes |  |  |  |
| Di Fabio | 1996 | yes |  |  |  |  |  |  | 1 | yes |  |  |  |
| Di | 2007 |  |  |  |  | yes |  |  | 1 | yes |  |  |  |
| Dinh | 2008 | yes |  | yes |  |  |  |  | 2 | Unclear |  |  |  |
| Dinnes | 2007 |  |  | yes | yes |  |  |  | 2 | yes |  | Total |  |
| Dinnes | 2003 | yes | yes |  |  |  |  |  | 2 | yes | yes | Total |  |
| Dong | 2008 | yes |  |  | yes |  |  |  | 2 | yes |  |  |  |
| Doria | 2006 | yes |  |  | yes |  |  |  | 2 | yes |  |  |  |
| Dubin | 2005 | yes |  |  | yes |  |  |  | 2 | yes |  |  |  |
| Ebell | 2004 | yes | yes |  |  |  |  |  | 2 | yes |  |  |  |
| Engelbrecht | 2002 |  |  |  | yes |  |  |  | 1 | yes |  |  |  |
| Ewald | 2004 |  |  | yes |  |  |  |  | 1 | no |  |  |  |
| Ewald | 2008 |  |  | yes |  |  |  |  | 1 | yes |  |  |  |
| Fancher | 2004 | yes | yes |  |  |  |  | yes | 3 | Unclear |  |  |  |
| Fischer | 2001 | yes | yes |  |  |  |  |  | 2 | Unclear |  |  |  |
| Flores | 2005 |  |  | yes | yes |  |  |  | 2 | yes |  |  |  |
| Ford | 2008 | yes | yes | yes | yes |  |  |  | 4 | yes |  |  |  |
| Fraquelli | 2005 | yes |  |  | yes |  |  |  | 2 | yes | yes |  |  |
| Friedrich-Rust | 2008 |  |  |  | yes |  |  |  | 1 | yes |  | Total |  |
| Geifman-Holtzman | 2006 |  |  |  |  |  |  | yes | 1 | no |  |  |  |
| Gisbert | 2006 | yes | yes |  |  |  |  |  | 2 | yes |  | Total |  |
| Glas | 2003 |  |  |  |  | yes |  |  | 1 | yes | yes |  |  |
| Goodacre | 2006 | yes | yes |  | yes |  | yes |  | 4 | yes |  |  |  |
| Gordon | 2003 |  | yes |  |  |  |  |  | 1 | yes |  |  |  |
| Goto | 2003 |  |  |  | yes |  |  |  | 1 | Unclear |  |  |  |
| Gould | 2003 |  |  | yes | yes |  |  |  | 2 | no |  |  |  |
| Gu | 2007 | yes |  |  |  |  |  |  | 1 | yes | yes | Partial |  |
| Gupta | 2002 |  | yes |  |  |  |  |  | 1 | yes |  |  |  |
| Hallan | 1997 |  |  |  | yes |  |  |  | 1 | yes |  |  |  |
| Halligan | 2005 |  |  |  |  |  | yes |  | 1 | yes |  | Partial |  |
| **Reference** | **Year** | **Pooled sen/spe** | **Pooled LR** | **Pooled DOR** | **SROC** | **Bivariate** | **HSROC** | **Other** | **Techniques per paper** | **Hetero- geneity** | **Correl- ation** | **Quadas** | **Quadas Score** |
| Hamon | 2008 | yes | yes | yes | yes |  |  |  | 4 | yes |  | Total |  |
| Hamon | 2008 | yes | yes | yes | yes |  |  |  | 4 | yes |  | Total |  |
| Hancock | 2007 | yes | yes |  | yes |  |  |  | 3 | no |  | Total | yes |
| Hayashino | 2005 | yes |  |  | yes |  |  |  | 2 | Unclear |  |  |  |
| Hegedus | 2007 | yes |  | yes | yes |  |  |  | 3 | yes |  | Total | yes |
| Hegedus | 2008 | yes |  | yes | yes |  |  |  | 3 | Unclear |  | Total | yes |
| Heijenbrok-Kal | 2007^a^ | yes |  | yes | yes |  |  |  | 3 | yes |  |  |  |
| Heijenbrok-Kal | 2007 ^b^ | yes |  |  | yes |  |  |  | 2 | no |  |  |  |
| Hobby | 2001 | yes |  |  |  |  |  |  | 1 | yes |  |  |  |
| Hofman | 2000 |  |  |  | yes |  |  |  | 1 | yes | yes |  |  |
| Holmes | 2007 | yes | yes |  |  |  |  |  | 2 | yes |  |  |  |
| Holroyd-Leduc | 2008 |  | yes |  |  |  |  |  | 1 | yes |  |  |  |
| Holty | 2005 | yes |  |  | yes |  |  |  | 2 | yes |  |  |  |
| Horsthuis | 2008 | yes | yes |  |  | yes |  |  | 3 | yes |  | Partial |  |
| Hovels | 2008 | yes | yes | yes | yes |  |  |  | 4 | Unclear |  |  |  |
| Huicho | 2002 |  |  |  | yes |  |  |  | 1 | yes |  |  |  |
| Ioannidis | 2003 | yes |  |  | yes |  |  |  | 2 | yes |  |  |  |
| Jahromi | 2005 | yes |  |  |  |  |  |  | 1 | yes |  |  |  |
| Jiang | 2007 | yes | yes | yes | yes |  |  |  | 4 | yes |  | Total | yes |
| Jones | 2005 | yes |  |  | yes |  |  |  | 2 | yes |  |  |  |
| Joshi | 2007 | yes |  |  |  |  |  |  | 1 | Unclear |  |  |  |
| Kalantri | 2005 |  |  |  | yes |  |  |  | 1 | yes |  | Partial |  |
| Karassa | 2006 | yes |  |  | yes |  |  |  | 2 | yes |  |  |  |
| Karassa | 2005 | yes |  |  | yes |  |  |  | 2 | yes |  |  |  |
| Kassai | 2004 | yes |  | yes | yes |  |  |  | 3 | yes |  |  |  |
| Kelly | 2001 |  |  |  | yes |  |  |  | 1 | Unclear |  |  |  |
| Khunti | 2004 |  |  |  | yes |  |  |  | 1 | yes |  |  |  |
| Koliopoulos | 2007 | yes |  |  |  |  |  |  | 1 | Unclear |  |  |  |
| Kraag | 1995 |  |  | yes |  |  |  |  | 1 | yes |  |  |  |
| Krug | 2008 | yes | yes | yes |  |  |  |  | 3 | yes |  | Total |  |
| Kwee | 2008 |  |  |  |  | yes |  |  | 1 | yes |  | Subs | yes |
| Kwee | 2007 |  |  |  |  | yes |  |  | 1 | yes |  | Total | yes |
| Lameris | 2008 |  |  |  |  | yes |  |  | 1 | yes |  | Total |  |
| Leal | 2008 | yes | yes | yes | yes |  |  |  | 4 | yes |  |  |  |
| Leeflang | 2008 |  |  |  | yes | yes |  |  | 2 | yes |  | Total |  |
| Liang | 2008^a^ | yes |  |  | yes |  |  |  | 2 | yes |  | Total | yes |
| **Reference** | **Year** | **Pooled sen/spe** | **Pooled LR** | **Pooled DOR** | **SROC** | **Bivariate** | **HSROC** | **Other** | **Techniques per paper** | **Hetero- geneity** | **Correl- ation** | **Quadas** | **Quadas Score** |
| Liang | 2008^b^ | yes |  |  | yes |  |  |  | 2 | yes |  | Total | yes |
| Ling | 2008^a^ | yes | yes | yes | yes |  |  |  | 4 | yes |  | Partial |  |
| Ling | 2008^b^ |  |  |  | yes | yes |  |  | 2 | yes |  | Partial |  |
| Liu | 2006 | yes |  | yes | yes |  |  |  | 3 | yes |  |  |  |
| Lysakowski | 2001 | yes |  |  |  |  |  |  | 1 | Unclear |  |  |  |
| Makrydimas | 2003 | yes |  |  | yes |  |  |  | 2 | yes |  |  |  |
| Mant | 2004 | yes | yes |  |  |  |  |  | 2 | yes |  |  |  |
| Martin | 2008 | yes |  |  | yes |  |  |  | 2 | no |  | Total | yes |
| Martin | 2007 |  |  |  | yes |  |  |  | 1 | Unclear |  | Total | yes |
| Martin | 2006 | yes |  | yes |  |  |  |  | 2 | no |  | Total |  |
| Marx | 2005 |  | yes |  |  |  |  |  | 1 | yes |  |  |  |
| Medeiros | 2005 | yes | yes |  |  |  |  |  | 2 | no | yes |  |  |
| Meijer | 2008 | yes |  |  | yes |  |  |  | 2 | yes |  |  |  |
| Meserve | 2008 |  |  |  | yes |  |  | yes | 2 | yes |  |  |  |
| Micames | 2007 | yes |  |  | yes |  |  |  | 2 | no | yes |  |  |
| Mijnhout | 2001 | yes |  | yes | yes |  |  |  | 3 | yes | yes |  |  |
| Mitchell | 2008 | yes |  |  |  |  |  |  | 1 | Unclear |  |  |  |
| Mol | 1998^a^ |  |  |  | yes |  |  |  | 1 | yes | yes |  |  |
| Mol | 1998^b^ |  |  |  | yes |  |  |  | 1 | yes | yes |  |  |
| Moles | 2002 |  |  | yes | yes |  |  |  | 2 | yes |  |  |  |
| Morgan | 2005 | yes | yes |  | yes |  |  |  | 3 | no |  | Partial |  |
| Morisson | 2008 | yes |  |  | yes |  |  |  | 2 | yes | yes |  |  |
| Mowatt | 2008 | yes | yes | yes | yes |  | yes |  | 5 | yes |  | Total |  |
| Mowatt | 2004 |  | yes |  |  |  |  |  | 1 | yes | yes | Total |  |
| Muchow | 2008 | yes |  |  |  |  |  |  | 1 | yes |  |  |  |
| Mulhall | 2005 | yes |  |  | yes |  |  |  | 2 | yes | yes |  |  |
| Nallamothu | 2001 | yes |  |  | yes |  |  |  | 2 | yes | yes |  |  |
| Nandalur | 2007 |  |  |  |  | yes |  |  | 1 | no |  | Subs | yes |
| Nandalur | 2008 | yes |  |  |  | yes |  |  | 2 | yes |  | Subs | yes |
| Nayak | 2006 |  |  |  | yes |  |  |  | 1 | yes |  |  |  |
| Niemann | 2008 | yes |  |  | yes |  |  |  | 2 | no |  |  |  |
| Noguchi | 2005 | yes | yes |  | yes |  |  |  | 3 | yes |  |  |  |
| Numans | 2004 | yes |  |  | yes |  |  |  | 2 | yes |  |  |  |
| Ogilvie | 2005 | yes |  | yes | yes |  |  |  | 3 | yes |  |  |  |
| Ola | 2003 |  | yes |  |  |  |  |  | 1 | no |  |  |  |
| Owens | 1996 |  |  |  | yes |  |  |  | 1 | yes |  |  |  |
| Pai | 2004 | yes | yes | yes | yes |  |  |  | 4 | yes |  |  |  |
| **Reference** | **Year** | **Pooled sen/spe** | **Pooled LR** | **Pooled DOR** | **SROC** | **Bivariate** | **HSROC** | **Other** | **Techniques per paper** | **Hetero- geneity** | **Correl- ation** | **Quadas** | **Quadas Score** |
| Pai | 2003 | yes | yes |  | yes |  |  |  | 3 | yes |  |  |  |
| Pai | 2005 | yes |  |  | yes |  |  |  | 2 | Unclear |  |  |  |
| Pai | 2007 |  |  |  | yes |  |  |  | 1 | Unclear |  | Partial |  |
| Pakos | 2005 | yes | yes |  | yes |  |  |  | 3 | no |  |  |  |
| Pakos | 2007^a^ | yes | yes | yes | yes |  | yes |  | 5 | yes |  | Total |  |
| Pakos | 2007^b^ | yes | yes |  | yes |  |  |  | 3 | yes |  |  |  |
| Patwardhan | 2004 | yes |  |  | yes |  |  |  | 2 | yes |  |  |  |
| Peters | 2008 | yes |  | yes | yes | yes |  |  | 4 | yes | yes | Partial |  |
| Pfeiffer | 2006 | yes |  |  | yes |  |  |  | 2 | yes |  |  |  |
| Pirozzo | 2003 |  |  |  | yes |  |  |  | 1 | Unclear |  |  |  |
| Price | 2005 | yes | yes | yes | yes |  |  |  | 4 | yes |  |  |  |
| Puli | 2008^a^ | yes | yes | yes | yes |  |  |  | 4 | Unclear |  |  |  |
| Puli | 2008^b^ | yes | yes | yes | yes |  |  |  | 4 | Unclear |  |  |  |
| Puli | 2008^c^ | yes | yes | yes | yes |  |  |  | 4 | Unclear |  |  |  |
| Puli | 2008^d^ | yes | yes |  | yes |  |  |  | 3 | no |  |  |  |
| Puli | 2007 | yes |  |  | yes |  |  |  | 2 | no |  |  |  |
| Purkayastha | 2007^a^ | yes |  | yes | yes |  |  |  | 3 | yes |  | Total | yes |
| Purkayastha | 2006 | yes |  |  | yes |  |  |  | 2 | yes |  |  |  |
| Purkayastha | 2005 | yes |  | yes | yes |  |  |  | 3 | yes |  |  |  |
| Purkayastha | 2007^b^ | yes |  | yes | yes |  |  |  | 3 | no |  | Total | yes |
| Reese | 2006 | yes | yes | yes | yes |  |  |  | 4 | yes |  | Total | yes |
| Roddam | 2005 |  |  |  | yes |  |  |  | 1 | yes |  |  |  |
| Rodgers | 2006 |  |  |  | yes |  |  |  | 1 | yes |  | Total |  |
| Ross | 2000 |  |  |  | yes |  |  |  | 1 | Unclear |  |  |  |
| Roy | 2005 |  | yes |  |  |  |  |  | 1 | no |  |  |  |
| Safdar | 2005 | yes |  |  | yes |  |  |  | 2 | yes |  |  |  |
| Samson | 2002 | yes |  |  | yes |  |  |  | 2 | Unclear |  |  |  |
| Sarmiento | 2003 | yes |  | yes | yes |  |  |  | 3 | Unclear |  |  |  |
| Sauerland | 2004 | yes |  |  | yes |  |  |  | 2 | yes |  |  |  |
| Scholten | 2001 | yes |  |  | yes |  |  |  | 2 | yes |  |  |  |
| Scholten | 2003 |  |  |  | yes | yes |  |  | 2 | Unclear | yes |  |  |
| Schreiber | 2003 | yes |  |  | yes |  |  |  | 2 | Unclear |  |  |  |
| Selman | 2005 | yes | yes |  |  |  |  |  | 2 | no |  | Partial |  |
| Selman | 2008^a^ | yes | yes |  |  | yes |  |  | 3 | no |  | Partial |  |
| Selman | 2008^b^ |  |  |  |  | yes |  |  | 1 | Unclear |  | Total |  |
| Shafiq | 2005 |  |  |  | yes |  |  |  | 1 | yes | yes |  |  |
| Shaheen | 2008 | yes |  | yes | yes | yes |  |  | 4 | no |  | Total | yes |
| **Reference** | **Year** | **Pooled sen/spe** | **Pooled LR** | **Pooled DOR** | **SROC** | **Bivariate** | **HSROC** | **Other** | **Techniques per paper** | **Hetero- geneity** | **Correl- ation** | **Quadas** | **Quadas Score** |
| Shaheen | 2007^a^ |  |  | yes | yes | yes |  |  | 3 | yes |  | Total | yes |
| Shaheen | 2007^b^ |  |  | yes | yes | yes |  |  | 3 | yes |  | Total |  |
| Shi | 2008 | yes |  |  | yes |  |  |  | 2 | yes |  | Total | yes |
| Shie | 2008 | yes |  |  | yes |  |  |  | 2 | yes |  |  |  |
| Shiga | 2006 | yes | yes | yes | yes |  |  |  | 4 | yes |  |  |  |
| Song | 2005 |  |  | yes |  |  |  |  | 1 | yes |  |  |  |
| Sosna | 2008 | yes |  |  |  |  |  |  | 1 | yes |  |  |  |
| Sotiriadis | 2003 | yes |  |  | yes |  |  |  | 2 | yes |  |  |  |
| Speight | 2006 | yes |  | yes | yes |  |  |  | 3 | yes |  |  |  |
| St John | 2006 | yes | yes | yes | yes |  |  |  | 4 | yes |  |  |  |
| Stein | 2006 | yes | yes |  |  |  |  |  | 2 | Unclear |  |  |  |
| Stein | 2004 | yes | yes |  |  |  |  | yes | 3 | no |  |  |  |
| Steingart | 2007^a^ |  |  |  | yes |  |  |  | 1 | yes |  | Partial |  |
| Steingart | 2007^b^ |  |  |  | yes |  |  |  | 1 | yes |  |  |  |
| Steingart | 2006 |  |  |  | yes |  |  |  | 1 | yes |  |  |  |
| Stengel | 2005 |  |  |  | yes |  |  |  | 1 | yes |  | Total | yes |
| Stengel | 2001 |  | yes |  | yes |  |  |  | 2 | yes |  |  |  |
| Takata | 2003 | yes |  |  |  |  |  |  | 1 | yes |  |  |  |
| Tang | 2007 | yes | yes | yes | yes |  |  |  | 4 | yes |  | Subs |  |
| Terasawa | 2004 | yes | yes |  |  |  |  |  | 2 | yes |  |  |  |
| Termaat | 2005 | yes |  |  |  |  |  |  | 1 | yes | yes |  |  |
| Tew | 2005 | yes |  |  | yes |  |  |  | 2 | yes |  |  |  |
| Trochez-Martinez | 2007 |  | yes | yes | yes |  |  |  | 3 | yes |  |  |  |
| Trowbridge | 2003 | yes | yes |  |  |  |  |  | 2 | Unclear |  |  |  |
| Tse | 2008 | yes |  | yes | yes |  |  |  | 3 | yes | yes | Partial |  |
| Tuon | 2006 | yes | yes | yes | yes |  |  |  | 4 | yes |  |  |  |
| Tuon | 2007 | yes |  | yes | yes |  |  |  |  | yes |  |  |  |
| Vakil | 2006 | yes | yes | yes | yes |  |  |  | 4 | yes |  |  |  |
| van Dongen | 2007 | yes | yes |  |  |  |  |  | 2 | yes | yes |  |  |
| van Randen | 2008 |  | yes |  |  | yes |  |  | 2 | Unclear |  | Partial |  |
| van Westreenen | 2004 |  |  |  |  | yes |  |  | 1 | Unclear |  |  |  |
| van Zaane | 2008 | yes |  |  |  | yes |  |  | 2 | yes |  | Total |  |
| Vanezis | 2008 | yes |  |  |  |  |  |  | 1 | Unclear |  |  |  |
| Vanhoenacker | 2007 | yes | yes | yes | yes |  |  |  | 4 | yes |  |  |  |
| Vasbinder | 2001 |  |  |  | yes |  |  |  | 1 | yes |  |  |  |
|  |  |  |  |  |  |  |  |  |  |  |  |  |  |
| **Reference** | **Year** | **Pooled sen/spe** | **Pooled LR** | **Pooled DOR** | **SROC** | **Bivariate** | **HSROC** | **Other** | **Techniques per paper** | **Hetero- geneity** | **Correl- ation** | **Quadas** | **Quadas Score** |
| Vestergaard | 2008 |  |  |  |  |  | yes |  | 1 | yes |  |  |  |
| Virgili | 2007 |  |  | yes | yes | yes |  |  | 3 | no |  | Total |  |
| Vlaar | 2007 |  |  | yes |  |  |  |  | 1 | no |  | Partial |  |
| von Roon | 2007 | yes |  | yes | yes |  |  |  | 3 | yes |  | Total | yes |
| Vroomen | 1999 | yes |  |  |  |  |  |  | 1 | Unclear |  |  |  |
| Wang | 2008 | yes |  |  | yes |  |  |  | 2 | yes |  |  |  |
| Wang | 2005 | yes |  | yes | yes |  |  |  | 3 | no |  |  |  |
| Wang | 2006 | yes | yes | yes | yes |  |  |  | 4 | yes |  | Partial |  |
| Wardlaw | 2006 | yes |  |  |  |  |  |  | 1 | no | yes | Subs |  |
| White | 2000 | yes |  |  | yes |  |  |  | 2 | yes |  |  |  |
| Whiting | 2006^a^ |  |  |  |  |  | yes |  | 1 | Unclear |  | Total |  |
| Whiting | 2006^b^ |  | yes |  | yes |  |  |  | 2 | yes |  | Total |  |
| Whitsel | 2000 | yes |  |  | yes |  |  |  | 2 | Unclear |  |  |  |
| Will | 2006 | yes |  | yes | yes |  |  |  | 3 | yes |  | Total | yes |
| Williams | 2007 |  |  |  | yes |  | yes |  | 2 | yes |  |  |  |
| Wittkampf | 2007 | yes |  |  |  |  |  |  | 1 | yes |  | Total |  |
| Worster | 2008 | yes | yes | yes | yes |  |  |  | 4 | yes | yes | Total | yes |
| Worster | 2002 |  | yes |  |  |  |  |  | 1 | no |  |  |  |
| Wykes | 2004 | yes | yes |  |  |  |  |  | 2 | yes | yes |  |  |

**Guide to table**

Pooled Sen/Spe – yes if study study reported on pooled sensitivity and pooled specificity.

Pooled LR – yes if study study reported on pooled likelihood ratios

Pooled DOR – yes if study reported on pooled diagnostic odds ratios

SROC – yes if study reported on SROC curve

Bivariate – yes if study reported on bivariate random effects model

HSROC – yes if study reported on a HSROC curve

Techniques per paper – number of statistical methods used to summarise data in paper

Heterogeneity – Study reported as present (yes), as not present (no), or not reported on ( unclear).

Correlation – study calculated a correlation coefficient.

Quadas – study implemented the QUADAS tool totally, substantially (8 or more items), or partially.

Quadas Score – study reported on an overall score.
