# Supplementary material for: Uptake of newer methodological developments and the deployment of meta-analysis in diagnostic test research: a systematic review
Source: BMC Med Res Methodol. 2011 Mar 14;11:27. doi: 10.1186/1471-2288-11-27 (PMC3065444; doi:10.1186/1471-2288-11-27)
Supplement: Additional file 6 — Appendix 6. Description of included studies. [file 1471-2288-11-27-S6.DOC]

**Appendix 6 – Description of the included studies**

| **Reference** | **Year** | **Diagnostic Test** | **Target disorder** | **Patient settings** | |
| --- | --- | --- | --- | --- | --- |
| Abdulla | 2007 | Radiology - CT coronary angiography - 64 slice multi-detector | IHD - Coronary artery disease > 50% stenosis | Cardiology | Cardiology |
| Abubakar | 2007 | Micro - Faecal - Rapid diagnostic Nucleic acid tests | Infection - Bacillus cereus in faeces | General medicine | Primary care |
|  |  | Micro - Faecal - Rapid diagnostic Nucleic acid tests | Infection - Campylobacter in faeces | Gastroenterology | Infectious diseases |
|  |  | Micro - Faecal - Rapid diagnostic Nucleic acid tests | Infection - Clostridium perfringens in faeces | General medicine | Primary care |
|  |  | Micro - Faecal - Rapid diagnostic Nucleic acid tests | Infection - Escherichia coli O157 in faeces | Gastroenterology | Infectious diseases |
|  |  | Micro - Faecal - Rapid diagnostic Nucleic acid tests | Infection - Salmonella spp in faeces | General medicine | Primary care |
|  |  | Micro - Faecal - Rapid diagnostic Nucleic acid tests | Infection - Staphylococcus aureus in faeces | Gastroenterology | Infectious diseases |
| Akcil | 2008 | Fine needle aspiration cytology of breast lumps | Cancer - Breast cancer | Surgery | Surgery |
|  |  | Biochem - Pleural fluid - adenosine deaminase | Infection - tuberculosis - Pleura - Brazil | Respiratory | Infectious diseases |
| Arbyn | 2008 | Cytology - Conventional cytology | Gynecol - CIN 2 | Gynaecology | Gynaecology |
|  |  | Cytology - Liquid base | Gynecol - CIN 2 | Gynaecology | Gynaecology |
| Arbyn | 2004 | Cytology - HPV HC test in patients with equivocal smear | Cancer - Cervical CIN | Gynaecology | Gynaecology |
|  |  | Cytology in patients with equivocal smear | Cancer - Cervical CIN | Gynaecology | Gynaecology |
| Atieh | 2008 | Micro - PCR | Infection - oral cavity - A actinomycetemcomitans | Dentistry | Dentistry |
|  |  | Micro - PCR | Infection - oral cavity - P. Ginigivalis | Dentistry | Dentistry |
| Bafounta | 2001 | Clinical - Dermoscopy | Cancer - Melanoma | Primary care | Dermatology |
|  |  | Clinical - examination | Cancer - Melanoma | Primary care | Dermatology |
| Bafounta | 2004 | Clinical - palpation | Cancer - melanoma metastasis in lymph nodes | Dermatology | Oncology |
|  |  | Radiology - Ultrasound | Cancer - melanoma metastasis in lymph nodes | Dermatology | Oncology |
| Bagai | 2006 | Clinical - Audioscope | Hearing impairment | ENT | Primary care |
|  |  | Clinical - do you have difficulty with hearing | Hearing impairment | ENT | Primary care |
|  |  | Clinical - HHIE-S questionnaire | Hearing impairment | ENT | Primary care |
|  |  | Clinical - Rinne tuning fork | Hearing impairment | Geriatrics | Primary care |
|  |  | Clinical - Whispered voice test | Hearing impairment | Geriatrics | Primary care |
| Bakis | 2004 | Cytology - Exfoliative by scalpel blade, swab or direct impression on a glass slide | Cancer - Basal cell carcinoma | Dermatology | Dermatology |
| Barnes | 2002 | Clinical - Pulse examination | Surgical arterial injury in traumatic knee injury | Emergency medicine | Orthopaedics |
| Bastian | 1998 | Bedside - Home pregnancy kits | Obstetric - Pregnancy | Obstetrics | Primary care |
| Benjaminse | 2006 | Clinical - Anterior drawer | Orthopaedic - Anterior cruciate ligament rupture | Emergency medicine | Orthopaedics |
|  |  | Clinical - Lachman test | Orthopaedic - Anterior cruciate ligament rupture | Emergency medicine | Orthopaedics |
|  |  | Clinical - Pivot shift test | Orthopaedic - Anterior cruciate ligament rupture | Emergency medicine | Orthopaedics |
| Berner | 2007 | Clinical - AUDIT test | Alcohol - At risk consumption | Primary care | Psychiatry |
| Berry | 1999 | Radiology - CT - electron beam | IHD - Coronary artery disase | Cardiology | Cardiology |
| Berry | 2002 | Radiology - MR - Angiography - Contrast enhanced | PVD - Carotid artery disease | General medicine | Vascular surgery |
|  |  | Radiology - MR - Angiography - phase Contrast | PVD - Carotid artery disease | Vascular surgery | Vascular surgery |
|  |  | Radiology - MR - Angiography - Time of flight (TOF) | PVD - Carotid artery disease | General medicine | Vascular surgery |
|  |  | Radiology - MR - Angiography - Contrast enhanced | PVD - Peripheral vascular disease | Vascular surgery | Vascular surgery |
|  |  | Radiology - MR - Angiography - phase Contrast | PVD - Peripheral vascular disease | General medicine | Vascular surgery |
|  |  | Radiology - MR - Angiography - Time of flight (TOF) | PVD - Peripheral vascular disease | Vascular surgery | Vascular surgery |
| Bipat | 2004 | Radiology - CT | Cancer - Rectal - Stage | Surgery | Oncology |
|  |  | Radiology - MR | Cancer - Rectal - Stage | Surgery | Oncology |
|  |  | Radiology - MR - coil | Cancer - Rectal - Stage | Surgery | Oncology |
|  |  | Radiology - MR - contrast | Cancer - Rectal - Stage | Surgery | Oncology |
|  |  | Radiology - Ultrasound - Endoluminal US, | Cancer - Rectal - Stage | Surgery | Oncology |
| Bipat | 2003 | Radiology - CT | Cancer - Uterine cervical carcinoma STAGING | Gynaecology | Oncology |
|  |  | Radiology - MR | Cancer - Uterine cervical carcinoma STAGING | Gynaecology | Oncology |
| Bipat | 2005 | Radiology - CT - abdomen | Cancer - Pancreatic adenocarcinoma | Surgery | Gastroenterology |
|  |  | Radiology - CT - Spiral - abdomen | Cancer - Pancreatic adenocarcinoma | Surgery | Gastroenterology |
|  |  | Radiology - MR - abdomen | Cancer - Pancreatic adenocarcinoma | Surgery | Gastroenterology |
|  |  | Radiology - Ultrasound - abdomen | Cancer - Pancreatic adenocarcinoma | Surgery | Gastroenterology |
| Blacksell | 2006 | Micro - Serum - Rapid immuno-chromatographic assays | Infection - Dengue | Infectious diseases | Infectious diseases |
| Brealey | 2005 | Radiology - X -rays - Radiographer reporting - All areas | Orthopaedic - Abnormalities on x-rays | Emergency medicine | Orthopaedics |
|  |  | Radiology - X -rays - Radiographer reporting - Whole skeleton | Orthopaedic - Abnormalities on x-rays | Emergency medicine | Orthopaedics |
|  |  | Radiology - X -rays - Radiographer reporting - Appendicular | Orthopaedic - Abnormalities on x-rays | Emergency medicine | Orthopaedics |
|  |  | Radiology - X -rays - Radiographer reporting - Axial | Orthopaedic - Abnormalities on x-rays | Emergency medicine | Orthopaedics |
| Brown | 2003 | Haem - Serum - D-dimer | T/E - Pulmonary embolism | General medicine | General medicine |
| Brown | 2002 | Haem - Serum - D-dimer - ELISA | T/E - Pulmonary embolism | General medicine | Respiratory |
| Bruyninckx | 2008 | Biochem - Serum - BNP | IHD - Heart failure - Left ventricular systolic dysfunction - Community setting screening | Primary care | Primary care |
|  |  | Biochem - Serum - NT-proBNP | IHD - Heart failure - Left ventricular systolic dysfunction - Community setting screening | Primary care | Primary care |
|  |  | Biochem - Serum - BNP | IHD - Heart failure - symptomatic patients | Cardiology | Emergency medicine |
|  |  | Biochem - Serum - NT-proBNP | IHD - Heart failure - symptomatic patients | Cardiology | Primary care |
| Burr | 2007 | Goldmann applanation tonometry | Open angle glaucoma - raised intraocular pressure | Ophthalmology | Ophthalmology |
|  |  | Non contact tonometry | Open angle glaucoma - raised intraocular pressure | Ophthalmology | Ophthalmology |
|  |  | Frequency doubling technology C20-1 | Open angle glaucoma -functional damage | Ophthalmology | Ophthalmology |
|  |  | Frequency doubling technology C20-5 | Open angle glaucoma -functional damage | Ophthalmology | Ophthalmology |
|  |  | Oculokinetic perimetry | Open angle glaucoma -functional damage | Ophthalmology | Ophthalmology |
|  |  | Radiology - Heidelberg retina tomograph | Open angle glaucoma -functional damage | Ophthalmology | Ophthalmology |
|  |  | Standard automated perimetry (supra threshold) | Open angle glaucoma -functional damage | Ophthalmology | Ophthalmology |
|  |  | Standard automated perimetry (threshold) | Open angle glaucoma -functional damage | Ophthalmology | Ophthalmology |
|  |  | Clinical - Ophthalmoscopy | Open angle glaucoma -structural damage | Ophthalmology | Ophthalmology |
|  |  | Optic disc photography | Open angle glaucoma -structural damage | Ophthalmology | Ophthalmology |
|  |  | Retinal nerve fibre layer photography | Open angle glaucoma -structural damage | Ophthalmology | Ophthalmology |
| Campens | 1997 | Biochem - Serum Creatinine | Renal - Glomerular filtration rate (GFR) | Nephrology | Paediatrics |
|  |  | Biochem - Serum Urea | Renal - Glomerular filtration rate (GFR) | Nephrology | Paediatrics |
|  |  | Biochem - Urine - 24hr Cr clearence | Renal - Glomerular filtration rate (GFR) | Nephrology | Paediatrics |
| Castilla-Rilo | 2007 | Clinical - IADL | Mental health - Dementia | Geriatrics | Psychiatry |
| Cavallazzi | 2008 | Biochem - Serum - BNP | Right ventricular dysfunction secondary to PE | Cardiology | Respiratory |
|  |  | Biochem - Serum - NT-proBNP | Right ventricular dysfunction secondary to PE | Cardiology | Respiratory |
| Cepoiu | 2008 | Clinical - Non-psychiatrist physician recognition of depression | Mental health - Depression | Primary care | Emergency medicine |
|  |  | Clinical - Non-psychiatrist physician recognition of depression - chart review | Mental health - Depression | Primary care | Emergency medicine |
| Chalco | 2005 | Clinical - conjunctival pallor | Anaemia in children | Paediatrics | Paediatrics |
|  |  | Clinical - nailbed pallor | Anaemia in children | Paediatrics | Paediatrics |
|  |  | Clinical - palmar pallor | Anaemia in children | Paediatrics | Paediatrics |
| Chen | 2001 | Clinical - Dermatologists diagnosis | Cancer - Melanoma | Primary care | Dermatology |
|  |  | Clinical - Primary care physicians diagnosis | Cancer - Melanoma | Primary care | Dermatology |
| Chua | 2008 | Radiology - CT angiography | Gastro/Surg - Gastrointestinal bleeding - source | Gastroenterology | Surgery |
| Clark | 2001 | Histology - Outpatient endometrial biopsy in women with abnormal uterine bleeding | Gynaecol - Endometrial hyperplasia | Gynaecology | Gynaecology |
| Clark | 2002 | Hysteroscopy - Premenopausal women | Cancer - Endometrial carcinoma | Gynaecology | Gynaecology |
|  |  | Hysteroscopy - Premenopausal women | Cancer - Endometrial carcinoma | Gynaecology | Gynaecology |
|  |  | Hysteroscopy - Postmenopausal women | Gynaecol - Endometrial hyperplasia | Gynaecology | Gynaecology |
|  |  | Hysteroscopy - Postmenopausal women | Gynaecol - Endometrial hyperplasia | Gynaecology | Gynaecology |
| Clark | 2000 | Clinical - Therapeutic trial - Acute Levo-DOPA | Parkinson's disease and parkinson's syndromes | Geriatrics | Neurology |
|  |  | Clinical - Therapeutic trial - Apomorphine challenge test | Parkinson's disease and parkinson's syndromes | Geriatrics | Neurology |
|  |  | Clinical - Therapeutic trial - Chronic Levo-DOPA | Parkinson's disease and parkinson's syndromes | Geriatrics | Neurology |
| Colin | 2001 | Micro - Serum - Hep C antibody test - 3rd generation | Infection - Hepatitis C | Infectious diseases | Gastroenterology |
| Dales | 1990 | Radiology - CT - contrast enhanced | Cancer - Lung - mediastinal lymph nodes to stage | Respiratory | Oncology |
|  |  | Radiology - CT - unenhanced | Cancer - Lung - mediastinal lymph nodes to stage | Respiratory | Oncology |
| de Bondt | 2007 | Radiology - CT | Cancer - SCC of the Head and Neck - lymph node metastases | ENT | Oncology |
|  |  | Radiology - MR | Cancer - SCC of the Head and Neck - lymph node metastases | ENT | Oncology |
|  |  | Radiology - Ultrasound | Cancer - SCC of the Head and Neck - lymph node metastases | ENT | Oncology |
|  |  | Radiology - Ultrasound guided fine needle aspiration | Cancer - SCC of the Head and Neck - lymph node metastases | ENT | Oncology |
| de Kroon | 2003 | Radiology - Saline contrast hysterosonography | Gynaecol - Uterine abnormatilies - fibroids | Gynaecology | Gynaecology |
|  |  | Radiology - Saline contrast hysterosonography | Gynaecol - Uterine abnormatilies - for abnormal uterine bleeding | Gynaecology | Gynaecology |
|  |  | Radiology - Saline contrast hysterosonography | Gynaecol - Uterine abnormatilies - intrauterine polyps | Gynaecology | Gynaecology |
| Debrey | 2008 | Radiology - MR - Angiography | PVD - Internal carotid artery disease - occlusion | Vascular surgery | Neurology |
|  |  | Radiology - MR - Angiography - Contrast enhanced | PVD - Internal carotid artery disease - occlusion | Vascular surgery | Neurology |
|  |  | Radiology - MR - Angiography - Contrast enhanced | PVD - Internal carotid artery disease - stenosis | Vascular surgery | Neurology |
|  |  | Radiology - MR - Angiography | PVD - internal carotid artery disease stenosis | Vascular surgery | Neurology |
| Delgado-Bolton | 2003 | Radiology - FDG-PET - Head & Neck - unknown primary tumour | Cancer - Primary tumour detection | Oncology | Oncology |
|  |  | Radiology - FDG-PET - Whole body - unknown primary tumours | Cancer - Primary tumour detection | Oncology | Oncology |
| Deville | 2000 | Clinical - Cross straight leg raising test | Orthopaedic - Herniated discs | Orthopaedics | Orthopaedics |
|  |  | Clinical - Lasegue test | Orthopaedic - Herniated discs | Orthopaedics | Orthopaedics |
| Deville | 2004 | Bedside - Urine - dipstick - both | Infection - Urinary tract infections | Primary care | General medicine |
|  |  | Bedside - Urine - dipstick - Leucocyte esterase | Infection - Urinary tract infections | Primary care | General medicine |
|  |  | Bedside - Urine - dipstick - Nitrite | Infection - Urinary tract infections | Primary care | General medicine |
| Di Fabio | 1996 | Platform Posturography | Benign Paroxysmal Positional Vertigo (BPPV) | ENT | Neurology |
|  |  | Platform Posturography | BPPV, | ENT | Neurology |
|  |  | Platform Posturography | Peripheral vestibular defects | ENT | Neurology |
| Di | 2007 | Haem - Serum - D-dimer | T/E - Deep Venous Thrombosis | General medicine | General medicine |
|  |  | Haem - Serum - D-dimer | T/E - Pulmonary embolism | General medicine | General medicine |
| Dinh | 2008 | Clinical - Probe to bone test | Infection - Osteomyelitis in diabetic foot ulcers | Endocrinology | Endocrinology |
|  |  | Radiology - Bone scan | Infection - Osteomyelitis in diabetic foot ulcers | Endocrinology | Endocrinology |
|  |  | Radiology - Leucocyte scan | Infection - Osteomyelitis in diabetic foot ulcers | Endocrinology | Endocrinology |
|  |  | Radiology - MR | Infection - Osteomyelitis in diabetic foot ulcers | Endocrinology | Endocrinology |
|  |  | Radiology - X-ray | Infection - Osteomyelitis in diabetic foot ulcers | Endocrinology | Endocrinology |
| Dinnes | 2007 | Micro - Serum - Anti-TB antibody tests | Infection - tuberculosis - Extrapulmonary | Infectious diseases | Respiratory |
|  |  | Micro - Sputum - Nucleic acid amplification test | Infection - tuberculosis - Extrapulmonary | Infectious diseases | Respiratory |
|  |  | Micro - Urine - Nucleic acid amplification test | Infection - tuberculosis - genito-urinary | Infectious diseases | Respiratory |
|  |  | Micro - Lymph - Nucleic acid amplification test | Infection - tuberculosis - lymphatic spread | Infectious diseases | Respiratory |
|  |  | Biochem - CSF - Adenosine deaminase | Infection - tuberculosis - Meningitis | Infectious diseases | Respiratory |
|  |  | Micro - CSF - anti-TB antibody test | Infection - tuberculosis - Meningitis | Infectious diseases | Respiratory |
|  |  | Micro - CSF - Nucleic acid amplification test | Infection - tuberculosis - Meningitis | Infectious diseases | Respiratory |
|  |  | Biochem - Pericardial fluid - Adenosine deaminase | Infection - tuberculosis - Pericardium | Infectious diseases | Respiratory |
|  |  | Micro - Pericardial fluid - Nucleic acid amplification test | Infection - tuberculosis - Pericardium | Infectious diseases | Respiratory |
|  |  | Biochem - Ascitic fluid - Adenosine deaminase | Infection - tuberculosis - Peritoneum | Infectious diseases | Respiratory |
|  |  | Biochem - Pleural fluid - adenosine deaminase | Infection - tuberculosis - Pleural | Infectious diseases | Respiratory |
|  |  | Biochem - Pleural fluid - Interferon gamma | Infection - tuberculosis - Pleural | Infectious diseases | Respiratory |
|  |  | Biochem - Pleural fluid - Interleukin | Infection - tuberculosis - Pleural | Infectious diseases | Respiratory |
|  |  | Biochem - Pleural fluid - Lysozyme | Infection - tuberculosis - Pleural | Infectious diseases | Respiratory |
|  |  | Biochem - Pleural fluid - TNF | Infection - tuberculosis - Pleural | Infectious diseases | Respiratory |
|  |  | Micro - Pleural fluid - anti-TB antibody test | Infection - tuberculosis - Pleural | Infectious diseases | Respiratory |
|  |  | Micro - Pleural fluid - nucliec acid amplification tests | Infection - tuberculosis - Pleural | Infectious diseases | Respiratory |
|  |  | Micro - Serum - Anti-TB antibody tests | Infection - tuberculosis - pulmonary | Infectious diseases | Respiratory |
|  |  | Micro - Sputum - Molecular amplification and probe tests | Infection - tuberculosis - pulmonary | Infectious diseases | Respiratory |
|  |  | Micro - Sputum - Nucleic acid amplification test | Infection - tuberculosis - pulmonary | Infectious diseases | Respiratory |
|  |  | Micro - Sputum - Phage tests | Infection - tuberculosis - pulmonary | Infectious diseases | Respiratory |
| Dinnes | 2003 | Radiology - MR | Orthopaedic - Shoulder - any thickness rotator cuff tear | Primary care | Emergency medicine |
|  |  | Radiology - MR - arthrography | Orthopaedic - Shoulder - any thickness rotator cuff tear | Primary care | Emergency medicine |
|  |  | Radiology - MR - fat suppressed | Orthopaedic - Shoulder - any thickness rotator cuff tear | Primary care | Emergency medicine |
|  |  | Radiology - Ultrasound | Orthopaedic - Shoulder - any thickness rotator cuff tear | Primary care | Emergency medicine |
|  |  | Clinical examination | Orthopaedic - Shoulder - Cause to shoulder pain | Orthopaedics | Emergency medicine |
|  |  | Radiology - MR | Orthopaedic - shoulder - full thickness rotator cuff tear | Orthopaedics | Emergency medicine |
|  |  | Radiology - MR - arthrography | Orthopaedic - shoulder - full thickness rotator cuff tear | Orthopaedics | Emergency medicine |
|  |  | Radiology - MR - fat suppressed | Orthopaedic - shoulder - full thickness rotator cuff tear | Orthopaedics | Emergency medicine |
|  |  | Radiology - Ultrasound | Orthopaedic - shoulder - full thickness rotator cuff tear | Orthopaedics | Primary care |
|  |  | Radiology - MR | Orthopaedic - Shoulder - partial thickness rotator cuff tear | Orthopaedics | Primary care |
|  |  | Radiology - MR - arthrography | Orthopaedic - Shoulder - partial thickness rotator cuff tear | Orthopaedics | Primary care |
|  |  | Radiology - MR - fat suppressed | Orthopaedic - Shoulder - partial thickness rotator cuff tear | Orthopaedics | Primary care |
|  |  | Radiology - Ultrasound | Orthopaedic - Shoulder - partial thickness rotator cuff tear | Orthopaedics | Primary care |
| Dong | 2008 | Radiology - FDG-PET/CT | Cancer - Unknown primary tumour | Oncology | Oncology |
|  |  | Radiology - FDG - PET | Cancer - Unknown primary tumour in patients with cervical LNs | Oncology | Oncology |
| Doria | 2006 | Radiology - CT of abdomen | Gastro/Surg - Appendicitis - Acute - Adults | Surgery | Surgery |
|  |  | Radiology - Ultrasound - abdomen | Gastro/Surg - Appendicitis - Acute - Adults | Surgery | Surgery |
|  |  | Radiology - CT of abdomen | Gastro/Surg - Appendicitis - Acute - Children | Surgery | Surgery |
|  |  | Radiology - Ultrasound - abdomen | Gastro/Surg - Appendicitis - Acute - Children | Surgery | Surgery |
| Dubin | 2005 | Micro - Swab - Middle meatal | Infection - Acute and Chronic sinusitis | ENT | ENT |
| Ebell | 2004 | Clinical - Chills | Infection - Influenza | Primary care | Primary care |
|  |  | Clinical - Cough | Infection - Influenza | Primary care | Primary care |
|  |  | Clinical - Headache | Infection - Influenza | Primary care | Primary care |
|  |  | Clinical - Myalgia | Infection - Influenza | Primary care | Primary care |
|  |  | Clinical - Nasal congestion | Infection - Influenza | Primary care | Primary care |
|  |  | Clinical - Nasal secretions | Infection - Influenza | Primary care | Primary care |
|  |  | Clinical - No sneezing | Infection - Influenza | Primary care | Primary care |
|  |  | Clinical - Not vaccinated | Infection - Influenza | Primary care | Primary care |
|  |  | Clinical - Objective temp | Infection - Influenza | Primary care | Primary care |
|  |  | Clinical - Oubjective temp | Infection - Influenza | Primary care | Primary care |
|  |  | Clinical - Sore throat | Infection - Influenza | Primary care | Primary care |
|  |  | Clinical - Sputum | Infection - Influenza | Primary care | Primary care |
| Engelbrecht | 2002 | Radiology - MR | Cancer - Prostate cancer - stage | Urology | Oncology |
|  |  | Radiology - MR - coil - endorectal | Cancer - Prostate cancer - stage | Urology | Oncology |
| Ewald | 2004 | Biochem - Urine - Alb/Cr ratio | Diabetes - Microalbuminuria | Primary care | Primary Care |
| Ewald | 2008 | Biochem - Serum - BNP | IHD - Heart failure - symptomatic patients - acute dyspnoea | Emergency medicine | Emergency medicine |
| Fancher | 2004 | Haem - Serum - D dimer - high sensitive | T/E - Deep Venous Thrombosis | General medicine | General medicine |
|  |  | Haem - Serum - D-dimer - SimpliRED | T/E - Deep Venous Thrombosis | General medicine | General medicine |
| Fischer | 2001 | Radiology - FDG - PET | Cancer - Lung - non-small cell - Staging | Respiratory | Oncology |
|  |  | Radiology - PET - Gamma camera PET | Cancer - Lung - non-small cell - Staging | Respiratory | Oncology |
| Flores | 2005 | Micro - Sputum - Nucleic acid amplification tests - in-house | Infection - Tuberculosis in sputa | Infectious diseases | Respiratory |
| Ford | 2008 | Clinical - abdominal mass | Cancer - Colorectal cancer | Emergency medicine | Surgery |
|  |  | Clinical - change of bowel habit | Cancer - Colorectal cancer | Emergency medicine | Surgery |
|  |  | Clinical - dark rectal bleeding | Cancer - Colorectal cancer | Emergency medicine | Surgery |
|  |  | Clinical - diarrhoea | Cancer - Colorectal cancer | Emergency medicine | Surgery |
|  |  | Clinical - rectal bleeding | Cancer - Colorectal cancer | Primary care | Gastroenterology |
|  |  | Clinical - weight loss | Cancer - Colorectal cancer | Primary care | Gastroenterology |
|  |  | Haem - Serum - anaemia | Cancer - Colorectal cancer | Primary care | Gastroenterology |
|  |  | Haem - Serum - Iron deficiency anaemia | Cancer - Colorectal cancer | Primary care | Gastroenterology |
| Fraquelli | 2005 | Radiology - Ultrasound - abdomen | Gastro/Surg - Inflammatory Bowel Disease - Crohn's | Gastroenterology | Gastroenterology |
| Friedrich-Rust | 2008 | Biochem - Pleural fluid - adenosine deaminase | Infection - tuberculosis - Pleura | Infectious diseases | Respiratory |
| Geifman-Holtzman | 2006 | Haem - Serum - maternal - for Fetal Rh genotyping | Obstetric - Fetal Rhesus status | Obstetrics | Obstetrics |
| Gisbert | 2006 | Micro - Faecal - Monoclonal stool antigen test for H Pylori | Infection - Helicobacter Pylori | Primary care | Gastroenterology |
| Glas | 2003 | Biochem - Urine - bladder tumour antigen (BTA) | Cancer - Primary bladder cancer | Urology | Urology |
|  |  | Biochem - Urine - BTA TRAK | Cancer - Primary bladder cancer | Urology | Urology |
|  |  | Biochem - Urine - FDP | Cancer - Primary bladder cancer | Urology | Urology |
|  |  | Biochem - Urine - NMP22 | Cancer - Primary bladder cancer | Urology | Urology |
|  |  | Biochem - Urine - telomerase | Cancer - Primary bladder cancer | Urology | Urology |
|  |  | Cytology - Urine - Cytology | Cancer - Primary bladder cancer | Urology | Urology |
| Goodacre | 2006 | Clinical - calf pain | T/E - Deep venous thrombosis - Proximal and calf | Primary care | Emergency medicine |
|  |  | Clinical - calf swelling | T/E - Deep venous thrombosis - Proximal and calf | Primary care | Emergency medicine |
|  |  | Clinical - Difference in calf diameter | T/E - Deep venous thrombosis - Proximal and calf | Primary care | Emergency medicine |
|  |  | Clinical - Erythema | T/E - Deep venous thrombosis - Proximal and calf | Primary care | Emergency medicine |
|  |  | Clinical - Homan's sign | T/E - Deep venous thrombosis - Proximal and calf | Primary care | General medicine |
|  |  | Clinical - Malignancy history | T/E - Deep venous thrombosis - Proximal and calf | Primary care | General medicine |
|  |  | Clinical - Obesity | T/E - Deep venous thrombosis - Proximal and calf | Primary care | General medicine |
|  |  | Clinical - Oedema | T/E - Deep venous thrombosis - Proximal and calf | Primary care | General medicine |
|  |  | Clinical - Past history of DVT | T/E - Deep venous thrombosis - Proximal and calf | Emergency medicine | General medicine |
|  |  | Clinical - Recent immobilisation | T/E - Deep venous thrombosis - Proximal and calf | Emergency medicine | General medicine |
|  |  | Clinical - Recent surgery | T/E - Deep venous thrombosis - Proximal and calf | Emergency medicine | General medicine |
|  |  | Clinical - Tenderness | T/E - Deep venous thrombosis - Proximal and calf | Emergency medicine | General medicine |
|  |  | Clinical - Warmth | T/E - Deep venous thrombosis - Proximal and calf | Emergency medicine | General medicine |
|  |  | Clinical - Wells score | T/E - Deep venous thrombosis - Proximal and calf | Primary care | General medicine |
|  |  | Haem - Serum - D-dimer | T/E - Deep venous thrombosis - Proximal and calf | Emergency medicine | General medicine |
|  |  | Plethysmography and rheography techniques | T/E - Deep venous thrombosis - Proximal and calf | Emergency medicine | General medicine |
|  |  | Radiology - CT | T/E - Deep venous thrombosis - Proximal and calf | Emergency medicine | General medicine |
|  |  | Radiology - MR | T/E - Deep venous thrombosis - Proximal and calf | Emergency medicine | General medicine |
|  |  | Radiology - Ultrasound | T/E - Deep venous thrombosis - Proximal and calf | Emergency medicine | General medicine |
| Gordon | 2003 | Radiology - Cystogram - Micturating | Renal parenchymal damage in children with proven UTI | Paediatrics | Urology |
| Goto | 2003 | Biochem - Pleural fluid - adenosine deaminase | Infection - Tuberculous pleurisy | Infectious diseases | Respiratory |
| Gould | 2003 | Radiology - CT | Cancer - Lung - non-small cell - mediastinal lymph nodes to stage | Oncology | Respiratory |
|  |  | Radiology - FDG - PET | Cancer - Lung - non-small cell - mediastinal lymph nodes to stage | Oncology | Respiratory |
| Gu | 2007 | Biochem - Pleural fluid - CEA | Cancer - Malignant pleural effusions | Respiratory | Oncology |
|  |  | Biochem - Pleural fluid - CYFRA 21-1 | Cancer - Malignant pleural effusions | Respiratory | Oncology |
| Gupta | 2002 | Radiology - Ultrasound - pelvic - post menopausal bleeding | Cancer - Uterine abnormality - Endometrial pathology carcinoma | Gynaecology | Gynaecology |
|  |  | Radiology - Ultrasound - pelvic - post menopausal bleeding | Gynaecol - Uterine abnormality - Endometrial hyperplasia | Gynaecology | Gynaecology |
| Hallan | 1997 | Biochem - Serum - CRP | Gastro/Surg - Appendicitis - acute | Surgery | Surgery |
|  |  | Haem - Serum - leucocyte count | Gastro/Surg - Appendicitis - acute | Surgery | Surgery |
| Halligan | 2005 | Radiology - CT - colonography | Cancer - Colorectal polyps & Cancer | Gastroenterology | Gastroenterology |
| Hamon | 2008 | Radiology - CT - Spiral - > 16 slice | IHD - Coronary stent - restenosis | Cardiology | Cardiology |
|  |  | Radiology - CT - Spiral - 16 slice | IHD - Coronary stent - restenosis | Cardiology | Cardiology |
| Hamon | 2008 | Radiology - CT - Spiral | IHD - Coronary artery bypass graft occlusion | Cardiology | Cardiology |
|  |  | Radiology - CT - Spiral | IHD - Coronary artery bypass graft stenosis | Cardiology | Cardiology |
|  |  | Radiology - CT - Spiral - 64 section | IHD - Coronary artery bypass graft stenosis | Cardiology | Cardiology |
|  |  | Radiology - CT - Spiral -16 section | IHD - Coronary artery bypass graft stenosis | Cardiology | Cardiology |
| Hancock | 2007 | Clinical - centralisation examination (SIJ), | Orthopaedic - Location of pathololgy in lower back pain - disc, facet, SIJ | Orthopaedics | Primary Care |
|  |  | Clinical - Spinous process vibration | Orthopaedic - Location of pathololgy in lower back pain - disc, facet, SIJ | Orthopaedics | Primary Care |
|  |  | Radiology - MR - disc degeneration | Orthopaedic - Location of pathololgy in lower back pain - disc, facet, SIJ | Orthopaedics | Primary Care |
|  |  | Radiology - MR - high intensity zone | Orthopaedic - Location of pathololgy in lower back pain - disc, facet, SIJ | Orthopaedics | Primary Care |
| Hayashino | 2005 | Radiology - CT - helical | T/E - Pulmonary embolism | Cardiology | Respiratory |
|  |  | Radiology - VQ scan | T/E - Pulmonary embolism | Cardiology | Respiratory |
| Hegedus | 2007 | Clinical Apley | Orthopaedic - Meniscal tear | Orthopaedics | Orthopaedics |
|  |  | Clinical - joint line tenderness | Orthopaedic - Meniscal tear | Orthopaedics | Orthopaedics |
|  |  | Clinical - McMurray | Orthopaedic - Meniscal tear | Orthopaedics | Orthopaedics |
| Hegedus | 2008 | Clinical - Speed test | Orhtopaedic - Shoulder - SLAP lesion | Orthopaedics | Emergency medicine |
|  |  | Clinical - Hawkins-Kennedy test | Orthopaedic - Shoulder - impingement | Orthopaedics | Primary care |
|  |  | Clinical - Neer test | Orthopaedic - Shoulder - impingement | Orthopaedics | Primary care |
| Heijenbrok-Kal | 2007^a^ | Radiology - CT - electron beam | IHD - Coronary artery disease > 50% stenosis | Cardiology | Cardiology |
|  |  | Radiology - SPECT (Single photon emission CT) - Stress - Chemical | IHD - Coronary artery disease > 50% stenosis | Cardiology | Cardiology |
|  |  | Radiology - SPECT (Single photon emission CT) - Stress - Exercise | IHD - Coronary artery disease > 50% stenosis | Cardiology | Cardiology |
|  |  | Radiology - Ultrasound - Echocardiogram - Stress - Chemical | IHD - Coronary artery disease > 50% stenosis | Cardiology | Cardiology |
|  |  | Radiology - Ultrasound - Echocardiogram - Stress - Exercise | IHD - Coronary artery disease > 50% stenosis | Cardiology | Cardiology |
| Heijenbrok-Kal | 2007^b^ | Radiology - CT angiography - Multi-detector | PVD - Peripheral vascular disease (>50%) - lower legs in symptomatic patients | Vascular surgery | Vascular surgery |
| Hobby | 2001 | Radiology - MR - wrist | Orthopaedic - Intrinsic carpal ligaments | Orthopaedics | Orthopaedics |
|  |  | Radiology - MR - wrist | Orthopaedic - Tears of traingular fibrocartilage, | Orthopaedics | Orthopaedics |
|  |  | Radiology - MR - wrist | Osteonecrosis of carpal bones | Orthopaedics | Orthopaedics |
| Hofman | 2000 | Radiology - X-ray - Plain skull x-ray | Mild head injury - GCS 13-15 | Emergency medicine | Neurosurgery |
| Holmes | 2007 | Radiology - Ultrasound - Abdomen of children with blunt trauma | Gastro/Surg - Intra-abdominal injury | Emergency medicine | Surgery |
|  |  | Radiology - Ultrasound - Abdomen of children with blunt trauma | Gastro/Surg - Intraperiotoneal fluid | Emergency medicine | Surgery |
| Holroyd-Leduc | 2008 | Clinical - coughing, sneezing, lifting, walking | Urology - Urinary incontinence - stress | Primary care | Gynaecology |
|  |  | Clinical - Overall clinical assessment | Urology - Urinary incontinence - stress | Primary care | Gynaecology |
|  |  | Clinical - Q tip test | Urology - Urinary incontinence - stress | Primary care | Gynaecology |
|  |  | Clinical - stress test | Urology - Urinary incontinence - stress | Primary care | Gynaecology |
|  |  | Clinical - strong & sudden urge to void leak before toilet | Urology - Urinary incontinence - urge | Primary care | Gynaecology |
| Holty | 2005 | Transbronchial needle aspiration | Cancer - Lung - non-small cell - mediastinal lymph nodes to stage | Respiratory | Oncology |
| Horsthuis | 2008 | Radiology - CT | Cancer - Cervical - Lymph node status | Gynaecology | Oncology |
|  |  | Radiology - MR | Cancer - Cervical - Lymph node status | Gynaecology | Oncology |
|  |  | Radiology - PET | Cancer - Cervical - Lymph node status | Gynaecology | Oncology |
|  |  | Sentinel node | Cancer - Cervical - Lymph node status | Gynaecology | Oncology |
| Hovels | 2008 | Clinical - absence of chest wall tenderness | IHD - Acute coronary syndrome - unselected population | Cardiology | Cardiology |
|  |  | Clinical - Epigastric pain | IHD - Acute coronary syndrome - unselected population | Cardiology | Cardiology |
|  |  | Clinical - Nausea & vomiting | IHD - Acute coronary syndrome - unselected population | Cardiology | Cardiology |
|  |  | Clinical - Pain in back | IHD - Acute coronary syndrome - unselected population | Cardiology | Cardiology |
|  |  | Clinical - Pain in left arm & or shoulder | IHD - Acute coronary syndrome - unselected population | Cardiology | Cardiology |
|  |  | Clinical - Sweating | IHD - Acute coronary syndrome - unselected population | Primary care | Cardiology |
|  |  | Clinical - Epigastric pain | IHD - Myocardial infarction - Acute | Primary care | Cardiology |
|  |  | Clinical - Nausea & vomiting | IHD - Myocardial infarction - Acute | Primary care | Cardiology |
|  |  | Clinical - Oppressive pain | IHD - Myocardial infarction - Acute | Primary care | Primary care |
|  |  | Clinical - Pain in back | IHD - Myocardial infarction - Acute | Primary care | Primary care |
|  |  | Clinical - Pain in left arm & or shoulder | IHD - Myocardial infarction - Acute | Primary care | Primary care |
|  |  | Clinical - Pain in neck | IHD - Myocardial infarction - Acute | Primary care | Primary care |
|  |  | Clinical - Pain in right arm & or shoulder | IHD - Myocardial infarction - Acute | Primary care | Primary care |
|  |  | Clinical - Sweating | IHD - Myocardial infarction - Acute | Primary care | Primary care |
|  |  | Clinical - absence of chest wall tenderness | IHD - Myocardial infarction - Acute - Unselected population | Primary care | Primary care |
|  |  | Clinical - Nausea & vomiting | IHD - Myocardial infarction - Acute - Unselected population | Primary care | Primary care |
|  |  | Clinical - Oppressive pain | IHD - Myocardial infarction - Acute - Unselected population | Primary care | Primary care |
|  |  | Clinical - Sweating | IHD - Myocardial infarction - Acute - Unselected population | Primary care | Primary care |
| Huicho | 2002 | Bedside - Urine - dipstick - Leucocyte esterase | Infection - Urinary tract infection | Paediatrics | Paediatrics |
|  |  | Bedside - Urine - dipstick - Nitrite | Infection - Urinary tract infection | Paediatrics | Paediatrics |
|  |  | Micro - Urine - Centifuged - Pyuria | Infection - Urinary tract infection | Paediatrics | Paediatrics |
|  |  | Micro - Urine - Centrifuged - Bacteriuria | Infection - Urinary tract infection | Paediatrics | Paediatrics |
|  |  | Micro - Urine - Centrifuged - Pyuria and bacteriuria | Infection - Urinary tract infection | Paediatrics | Paediatrics |
| Ioannidis | 2003 | Radiology - FDG - PET | Cancer - Soft tissue sarcoma and staging | Oncology | Neurology |
| Jahromi | 2005 | Radiology - Ultrasound - Colour duplex | PVD - Internal carotid artery stenosis | Vascular surgery | Vascular surgery |
| Jiang | 2007 | Biochem - Pleural fluid - Interferon gamma | Infection - Tuberculous pleurisy | Respiratory | Infectious diseases |
| Jones | 2005 | Radiology - Ultrasound - Echocardiogram - Doppler | IHD - Left internal thoracic artery graft stenosis | Cardiology | Surgery |
| Joshi | 2007 | Radiology - FDG-PET - Attenuated corrected (AC) | Cancer lesion - not clear in oncology patients | Oncology | Oncology |
|  |  | Radiology - FDG-PET - Non attenuated | Cancer lesion - not clear in oncology patients | Oncology | Oncology |
| Kalantri | 2005 | Micro - Sputum - Bacteriaphage tests | Infection - Tuberculosis - in clinical specimens | Infectious diseases | Infectious diseases |
|  |  | Micro - Sputum - Microscopy | Infection - Tuberculosis - in clinical specimens | Infectious diseases | Infectious diseases |
|  |  | Micro - Sputum - Bacteriaphage tests | Infection - Tuberculosis - smear negative | Infectious diseases | Infectious diseases |
|  |  | Micro - Sputum - Bacteriaphage tests | Infection - Tuberculosis - smear positive | Cardiology | Cardiology |
| Karassa | 2006 | Biochem - Serum - Anti-robosomal P Protein antibodyusing Western blot or ELISA | SLE - Neuropsychiatric - Diffuse | Rheumatology | Rheumatology |
|  |  | Biochem - Serum - Anti-robosomal P Protein antibodyusing Western blot or ELISA | SLE - Neuropsychiatric - Focal neurology | Rheumatology | Rheumatology |
|  |  | Biochem - Serum - Anti-robosomal P Protein antibodyusing Western blot or ELISA | SLE - Neuropsychiatric - Overall | Rheumatology | Rheumatology |
|  |  | Biochem - Serum - Anti-robosomal P Protein antibodyusing Western blot or ELISA | SLE - Neuropsychiatric - Psychosis | Rheumatology | Rheumatology |
| Karassa | 2005 | Radiology - Ultrasound - Temporal artery | Giant cell arteritis | Rheumatology | Geriatrics |
| Kassai | 2004 | Radiology - Ultrasound - Duplex | T/E - Deep venous thrombosis - Calf in asymptomatic patients | General medicine | General medicine |
|  |  | Radiology - Ultrasound - Duplex | T/E - Deep venous thrombosis - Proximal in asymptomatic patients | General medicine | General medicine |
| Kelly | 2001 | Radiology - Ultrasound - Endoscopic | Cancer - Gastric - Stage | Gastroenterology | Oncology |
|  |  | Radiology - Ultrasound - Endoscopic | Cancer - Gastro -oesophageal - Stage | Gastroenterology | Oncology |
|  |  | Radiology - Ultrasound - Endoscopic | Cancer - Oesophageal - Stage | Gastroenterology | Oncology |
| Khunti | 2004 | ECG - 12 lead screening | IHD - Heart Failure - Left ventricular systolic dysfunction | Primary care | Primary care |
| Koliopoulos | 2007 | Cytology - cervical | Cancer - Pre- cancerous High grade CIN 2 | Primary care | Gynaecology |
|  |  | Cytology - cervical - HPV test - HC1 | Cancer - Pre- cancerous High grade CIN 2 | Primary care | Gynaecology |
|  |  | Cytology - cervical - HPV test - HC2 | Cancer - Pre- cancerous High grade CIN 2 | Primary care | Gynaecology |
|  |  | Cytology - cervical - PCR | Cancer - Pre- cancerous High grade CIN 2 | Primary care | Gynaecology |
| Kraag | 1995 | Clinical - abdominal pain | Gastro/Surg - Gall stones | Gastroenterology | Primary care |
|  |  | Clinical - belching | Gastro/Surg - Gall stones | Surgery | Emergency medicine |
|  |  | Clinical - Biliary pain | Gastro/Surg - Gall stones | Gastroenterology | Primary care |
|  |  | Clinical - bloating | Gastro/Surg - Gall stones | Surgery | Emergency medicine |
|  |  | Clinical - dyspepsia | Gastro/Surg - Gall stones | Gastroenterology | Primary care |
|  |  | Clinical - Epigastric or upper abdominal pain | Gastro/Surg - Gall stones | Surgery | Emergency medicine |
|  |  | Clinical - fat intolerance | Gastro/Surg - Gall stones | Gastroenterology | Primary care |
|  |  | Clinical - flatulence | Gastro/Surg - Gall stones | Surgery | Emergency medicine |
|  |  | Clinical - food intolerance | Gastro/Surg - Gall stones | Gastroenterology | Primary care |
|  |  | Clinical - heartburn | Gastro/Surg - Gall stones | Surgery | Emergency medicine |
|  |  | Clinical - nausea and vomiting | Gastro/Surg - Gall stones | Gastroenterology | Primary care |
|  |  | Clinical - pain constant | Gastro/Surg - Gall stones | Surgery | Emergency medicine |
|  |  | Clinical - pain in distinct attacks | Gastro/Surg - Gall stones | Gastroenterology | Primary care |
|  |  | Clinical - pain provoked by food | Gastro/Surg - Gall stones | Surgery | Emergency medicine |
|  |  | Clinical - pain radiation | Gastro/Surg - Gall stones | Gastroenterology | Primary care |
|  |  | Clinical - Right upper quadrant pain | Gastro/Surg - Gall stones | Surgery | Emergency medicine |
| Krug | 2008 | Radiology - FDG - PET | Cancer - melanoma - Stage - Overall | Dermatology | Oncology |
| Kwee | 2008 | Radiology - FDG - PET | Infection - Prosthetic joint infection | Orthopaedics | Orthopaedics |
| Kwee | 2007 | Radiology - MR - Angiography - Contrast enhanced | PVD - intra-cranial aneurysm with coil insertion - residual anurysmal flow within the aneurysm neck | Neurosurgery | Neurosurgery |
|  |  | Radiology - MR - Angiography - Time of flight (TOF) | PVD - intra-cranial aneurysm with coil insertion - residual anurysmal flow within the aneurysm neck | Neurosurgery | Neurosurgery |
| Lameris | 2008 | Radiology - CT | Gastro/Surg - Diverticulitis - Acute | Gastroenterology | Gastroenterology |
|  |  | Radiology - Ultrasound - Graded compression | Gastro/Surg - Diverticulitis - Acute | Gastroenterology | Gastroenterology |
| Leal | 2008 | Micro - Saliva - Antibody based detection test - ELISA | Infection - Helicobacter Pylori in children | Paediatrics | Paediatrics |
|  |  | Micro - Serum - Antibody based detection test - ELISA commercial | Infection - Helicobacter Pylori in children | Paediatrics | Paediatrics |
|  |  | Micro - Serum - Antibody based detection test - ELISA in house | Infection - Helicobacter Pylori in children | Paediatrics | Paediatrics |
|  |  | Micro - Serum - Antibody based detection test - Western blot | Infection - Helicobacter Pylori in children | Paediatrics | Paediatrics |
| Leeflang | 2008 | Biochem - Serum Galactomannan ELISA - Platelia - adults | Infection - Invasive aspergillosis in the immunocompromised | Respiratory | Infectious diseases |
|  |  | Biochem - Serum Galactomannan ELISA - Platelia - children | Infection - Invasive aspergillosis in the immunocompromised | Respiratory | Infectious diseases |
| Liang | 2008^a^ | Biochem - Pleural fluid - CA 125 | Cancer - Malignant pleural effusion | Respiratory | Oncology |
|  |  | Biochem - Pleural fluid - CA 125 & CYFRA 21-1 | Cancer - Malignant pleural effusion | Respiratory | Oncology |
|  |  | Biochem - Pleural fluid - CA 15-3 | Cancer - Malignant pleural effusion | Respiratory | Oncology |
|  |  | Biochem - Pleural fluid - CA 15-3 & CYFRA 21-1 | Cancer - Malignant pleural effusion | Respiratory | Oncology |
|  |  | Biochem - Pleural fluid - CA19-9 | Cancer - Malignant pleural effusion | Respiratory | Oncology |
|  |  | Biochem - Pleural fluid - CEA & CA 125 | Cancer - Malignant pleural effusion | Respiratory | Oncology |
|  |  | Biochem - Pleural fluid - CEA & CA 15-3 | Cancer - Malignant pleural effusion | Respiratory | Oncology |
|  |  | Biochem - Pleural fluid - CEA & CYFRA 21-1 | Cancer - Malignant pleural effusion | Respiratory | Oncology |
|  |  | Biochem - Pleural fluid - CYFRA 21-1 | Cancer - Malignant pleural effusion | Respiratory | Oncology |
| Liang | 2008^b^ | Radiology - CT | Gastro/Surg - Inflammatory bowel disease | Gastroenterology | Surgery |
|  |  | Radiology - MR | Gastro/Surg - Inflammatory bowel disease | Gastroenterology | Surgery |
|  |  | Radiology - SPECT | Gastro/Surg - Inflammatory bowel disease | Gastroenterology | Surgery |
|  |  | Radiology - Ultrasound | Gastro/Surg - Inflammatory bowel disease | Gastroenterology | Surgery |
| Ling | 2008^a^ | Micro - Sputum - Nucleic acid amplification tests - Commercial | Infection - tuberculosis - Pulmonary (results to 7 commercial kits) | Respiratory | Infectious diseases |
| Ling | 2008^b^ | Micro - GenoType MTBR assays | Infection - tuberculosis - isoniazid resistant TB | Infectious diseases | Respiratory |
|  |  | Micro - GenoType MTBR assays | Infection - tuberculosis - rifampicin resistant TB | Infectious diseases | Respiratory |
| Liu | 2006 | Clinical - Alvarado score | Gastro/Surg - Appendicitis - acute | General medicine | Gastroenterology |
|  |  | Clinical - Discrimination rule | Gastro/Surg - Appendicitis - acute | General medicine | Gastroenterology |
|  |  | Clinical - Neural network | Gastro/Surg - Appendicitis - acute | Emergency medicine | Surgery |
|  |  | Clinical decision tools naïve bayesian | Gastro/Surg - Appendicitis - acute | Emergency medicine | Surgery |
|  |  | Clinical logistic regression | Gastro/Surg - Appendicitis - acute | General medicine | Gastroenterology |
| Lysakowski | 2001 | Radiology - Ultrasound - Transcranial Doppler in ruptured cerebral aneurysm | Vasospasm - anterior cerebral artery | Neurosurgery | Neurology |
|  |  | Radiology - Ultrasound - Transcranial Doppler in ruptured cerebral aneurysm | Vasospasm - middle cerebral artery | Neurosurgery | Neurology |
| Makrydimas | 2003 | Radiology - Ultrasound - Foetal - nuchal translucency | Obstetric - Foetal - Congenital cardiac defects in chromosomally normal foetuses | Obstetrics | Obstetrics |
| Mant | 2004 | ECG - Abnormal ST segment and T wave | IHD - Angina - Exertional angina | Cardiology | General medicine |
|  |  | ECG - Exercise - Combinations | IHD - Angina - Exertional angina | Cardiology | General medicine |
|  |  | ECG - Exercise - ST depression | IHD - Angina - Exertional angina | Primary care | Emergency medicine |
|  |  | ECG - Exercise - ST slope | IHD - Angina - Exertional angina | Primary care | Emergency medicine |
|  |  | ECG - Q wave | IHD - Angina - Exertional angina | Primary care | Emergency medicine |
|  |  | ECG - Q wave or ST changes | IHD - Angina - Exertional angina | Cardiology | General medicine |
|  |  | Clinical - Central pain | IHD - Myocardial infarction | Cardiology | General medicine |
|  |  | Clinical - Crushing pain | IHD - Myocardial infarction | Primary care | Emergency medicine |
|  |  | Clinical - nausea and vomiting | IHD - Myocardial infarction | Primary care | Emergency medicine |
|  |  | Clinical - Pain on palpation | IHD - Myocardial infarction | Primary care | Emergency medicine |
|  |  | Clinical - Pleuritic pain | IHD - Myocardial infarction | Cardiology | General medicine |
|  |  | Clinical - Positional pain | IHD - Myocardial infarction | Cardiology | General medicine |
|  |  | Clinical - previous MI/angina | IHD - Myocardial infarction | Primary care | Emergency medicine |
|  |  | Clinical - radiation - any | IHD - Myocardial infarction | Primary care | Emergency medicine |
|  |  | Clinical - radiation - left side | IHD - Myocardial infarction | Primary care | Emergency medicine |
|  |  | Clinical - radiation - right side | IHD - Myocardial infarction | Cardiology | General medicine |
|  |  | Clinical - sharp pain | IHD - Myocardial infarction | Cardiology | General medicine |
|  |  | Clinical - sweating | IHD - Myocardial infarction | Primary care | Emergency medicine |
|  |  | ECG - normal | IHD - Myocardial infarction | Primary care | Emergency medicine |
|  |  | ECG - ST depression | IHD - Myocardial infarction | Primary care | Emergency medicine |
|  |  | ECG - ST elevation | IHD - Myocardial infarction | Primary care | Emergency medicine |
|  |  | ECG - ST elevation/ ST depression/Q/T | IHD - Myocardial infarction | Primary care | Emergency medicine |
|  |  | Clinical - Crushing pain | IHD - Unstable angina | Primary care | Emergency medicine |
|  |  | Clinical - radiation - left side | IHD - Unstable angina | Cardiology | General medicine |
| Martin | 2008 | Micro - Sputum - Nitrate reductase assay | Infection - Tuberculosis - isoniazid resisitance | Infectious diseases | Respiratory |
|  |  | Micro - Sputum - Nitrate reductase assay | Infection - Tuberculosis - rifampicin resistance | Infectious diseases | Respiratory |
| Martin | 2007 | Micro - Coloureimetric redox-indicator test | Infection - Tuberculosis - Isoniazid resistant | Infectious diseases | Infectious diseases |
|  |  | Micro - Coloureimetric redox-indicator test | Infection - Tuberculosis - Rifampicin resistant | Infectious diseases | Infectious diseases |
| Martin | 2006 | Clinical - Diary | Urology - urinary incontinence - type | Gynaecology | Urology |
|  |  | Clinical - history | Urology - urinary incontinence - type | Gynaecology | Urology |
|  |  | Clinical - Pad test | Urology - urinary incontinence - type | Gynaecology | Urology |
|  |  | Clinical - Q tip | Urology - urinary incontinence - type | Gynaecology | Urology |
|  |  | Clinical - Validated scale | Urology - urinary incontinence - type | Gynaecology | Urology |
|  |  | Radiology - Ultrasound | Urology - urinary incontinence - type | Gynaecology | Urology |
|  |  | Radiology - X-ray | Urology - urinary incontinence - type | Gynaecology | Urology |
|  |  | Stress test | Urology - urinary incontinence - type | Gynaecology | Urology |
|  |  | Urethral pressure | Urology - urinary incontinence - type | Gynaecology | Urology |
| Marx | 2005 | Bedside - Serum - finger prick - Parasite LDH rapid malaria tests | Infection - Malaria - P falciparum | Infectious diseases | Infectious diseases |
|  |  | Bedside - Serum - finger prick -HRP-2 rapid malaria tests | Infection - Malaria - P falciparum | Infectious diseases | Infectious diseases |
|  |  | Bedside - Serum - finger prick - Parasite LDH rapid malaria tests | Infection - Malaria - P vivax | Infectious diseases | Infectious diseases |
|  |  | Bedside - Serum - finger prick -HRP-2 & aldolase rapid malaria tests | Infection - Malaria - P vivax | Infectious diseases | Infectious diseases |
|  |  | Bedside - Serum - finger prick - Parasite LDH rapid malaria tests | Infection - Malaria - P. malariae & P. ovale | Infectious diseases | Infectious diseases |
|  |  | Bedside - Serum - finger prick -HRP-2 & aldolase rapid malaria tests | Infection - Malaria - P. malariae & P. ovale | Infectious diseases | Infectious diseases |
| Medeiros | 2005 | Histology - Frozen section analysis during surgery | Cancer - Ovarian - Borderline | Gynaecology | Gynaecology |
|  |  | Histology - Frozen section analysis during surgery | Cancer - Ovarian - Malignant | Gynaecology | Gynaecology |
| Meijer | 2008 | Radiology - CT angiography - Multidetector 40 slice | IHD - Coronary artery stenosis | Cardiology | Cardiology |
|  |  | Radiology - CT angiography - Multidetector 64 slice | IHD - Coronary artery stenosis | Cardiology | Cardiology |
| Meserve | 2008 | Clinical - Apley's test | Orthopaedic - Meniscal injury | Primary care | Orthopaedics |
|  |  | Clinical - joint line tenderness | Orthopaedic - Meniscal injury | Primary care | Orthopaedics |
|  |  | Clinical - McMurray | Orthopaedic - Meniscal injury | Primary care | Orthopaedics |
| Micames | 2007 | Radiology - Ultrasound - Endoscopic Ultrasound guided fine needle aspiration of mediastinal lymph nodes | Cancer - Lung - Non-small cell lung cancer staging - identifying metastasis | Oncology | Respiratory |
| Mijnhout | 2001 | Radiology - FDG-PET in melanoma patients | Cancer - Melanoma - Stage | Oncology | Dermatology |
| Mitchell | 2008 | Clinical - Subjective memory complaints | Mental health - Dementia | Geriatrics | Primary care |
|  |  | Clinical - Subjective memory complaints | Mental health - Mild cognitive impairment | Psychiatry | Primary care |
| Mol | 1998^a^ | Biochem - Serum - CA125 | Gynaecol - Endometriosis | Gynaecology | Gynaecology |
| Mol | 1998^b^ | Biochem - Serum progesterone | Obstetric - Pregnancy - Ectopic | Gynaecology | Gynaecology |
|  |  | Biochem - Serum progesterone | Obstetric - Pregnancy - Non viable | Gynaecology | Gynaecology |
| Moles | 2002 | Clinical - Systematic visual screening of oral cavity | Cancer - Oral cancer | ENT | Primary care |
| Morgan | 2005 | Micro - Culture - LiPA probe assay | Infection - Tuberculosis - Rifampicin resistant | Infectious diseases | Infectious diseases |
| Morisson | 2008 | Radiology - Ultrasound - FibroScan - transient elastography | Gastro/Surg - Liver fibrosis - Stage - cirrhosis | Gastroenterology | Hepatology |
|  |  | Radiology - Ultrasound - FibroScan - transient elastography | Gastro/Surg - Liver fibrosis - Stage - severe | Gastroenterology | Hepatology |
| Mowatt | 2008 | Radiology - CT angiography - 64-slice | IHD - Coronary artery disease > 50% stenosis | Cardiology | Cardiology |
| Mowatt | 2004 | ECG - Stress | IHD - Coronary artery disease > 50% stenosis | Cardiology | Cardiology |
|  |  | Radiology - SPECT myocardial perfusion scintography | IHD - Coronary artery disease > 50% stenosis | Cardiology | Cardiology |
| Muchow | 2008 | Radiology - MR - C-spine | Orthopaedic - Cervical spine injury in blunt trauma | Emergency medicine | Emergency medicine |
| Mulhall | 2005 | Radiology - CT - colonography | Gastro/surg - Colorectal Polyps | Gastroenterology | Gastroenterology |
| Nallamothu | 2001 | Radiology - CT - electron beam | IHD - Coronary artery disease - > 50% stenosis | Cardiology | Cardiology |
| Nandalur | 2007 | Radiology - MR - Stress induced wall motion imaging Cardiac test | IHD - Coronary artery disease > 50% stenosis | Cardiology | Cardiology |
|  |  | Radiology - MR - Stress perfusion imaging Cardiac test | IHD - Coronary artery disease > 50% stenosis | Cardiology | Cardiology |
| Nandalur | 2008 | Radiology - Ultrasound - Endoscopic | Cancer - oesophageal cancer - stage (did separate analyses for T1-T4, N stage) | Gastroenterology | Oncology |
|  |  | Radiology - Ultrasound - Endoscopic - FNA | Cancer - oesophageal cancer - stage (did separate analyses for T1-T4, N stage) | Gastroenterology | Oncology |
| Nayak | 2006 | Radiology - Calcaneal quantitative ultrasound | Osteoporosis as defined by DXA T scores of <-2.5 at either the hip or spine | Rheumatology | Rheumatology |
| Niemann | 2008 | Radiology - CT - Low dose | Renal stones | Urology | Urology |
| Noguchi | 2005 | Radiology - Ultrasound - Transesophageal echocardiogram - stress - transatrial pacing | IHD - Coronary artery disase | Cardiology | Cardiology |
|  |  | Radiology - Ultrasound - Echocardiogram - stress - chemical | IHD - Coronary artery disase | Cardiology | Cardiology |
|  |  | Radiology - Ultrasound - Echocardiogram - stress - Exercise | IHD - Coronary artery disase | Cardiology | Cardiology |
|  |  | Radiology - Ultrasound - Echocardiogram - stress - transatrial pacing | IHD - Coronary artery disase | Cardiology | Cardiology |
| Numans | 2004 | Clinical - Therapeutic trial - Proton pump inhibitor | Gastro/Surg - GORD | Gastroenterology | Gastroenterology |
| Ogilvie | 2005 | Micro - Self collected vaginal specimens | Infection - HPV | Primary care | Gynaecology |
| Ola | 2003 | Cytology - Sperm - In vitro penetration of cervical mucous | Sperm motility | Gynaecology | Gynaecology |
| Owens | 1996 | Micro - PCR | Infection - HIV in adults | Infectious diseases | Infectious diseases |
| Pai | 2004 | Micro - Nucleic acid amplification tests - in house | Infection - Tuberculous pleuritis | Infectious diseases | Respiratory |
|  |  | Micro - Nucleic acid amplification tests -commercial | Infection - Tuberculous pleuritis | Infectious diseases | Respiratory |
| Pai | 2003 | Micro - CSF - Nucleic acid amplification test - commercial | Infection - Tuberculous meningitis | Primary care | Infectious diseases |
|  |  | Micro - CSF - Nucleic acid amplification test - in house | Infection - Tuberculous meningitis | Infectious diseases | Infectious diseases |
| Pai | 2005 | Micro - Culture - Rapid bacteriophage assays - Commercial | Infection - Tuberculosis - Rifampicin resistance | Infectious diseases | Infectious diseases |
|  |  | Micro - Culture - Rapid bacteriophage assays - In-house | Infection - Tuberculosis - Rifampicin resistance | Infectious diseases | Infectious diseases |
| Pai | 2007 | Micro - Serum - Rapid HIV testing | Infection - HIV in pregnant women | Obstetrics | Obstetrics |
| Pakos | 2005 | Radiology - FDG - PET | Cancer - Lymphoma - Hodgkins - Bone marrow infiltration | Oncology | Haematology |
|  |  | Radiology - FDG - PET | Cancer - Lymphoma - Non-Hodgkins - Bone marrow infiltration | Oncology | Haematology |
| Pakos | 2007^a^ | Radiology - Scintography - antigranulocyte with monoclonal antibodies labeled with Tc-99 | Infection - Osteomyelitis | Rheumatology | Orthopaedics |
| Pakos | 2007^b^ | Radiology - Scintography - antigranulocyte with monoclonal antibodies labeled with Tc- 99 | Infection - Prosthesis - Hip | Orthopaedics | Orthopaedics |
|  |  | Radiology - Scintography - antigranulocyte with monoclonal antibodies labeled with Tc- 99 | Infection - Prosthesis - Knee | Orthopaedics | Orthopaedics |
| Patwardhan | 2004 | Radiology - FDG - PET | Mental Health - Alzheimers disease | Psychiatry | Geriatrics |
| Peters | 2008 | Radiology - MR - Contrast enhanced of breast | Cancer - Breast cancer in those with breast lesions | Surgery | Oncology |
| Pfeiffer | 2006 | Biochem - Serum - Galactomannan assay | Infection -Invasive aspergillosis | Infectious diseases | Infectious diseases |
| Pirozzo | 2003 | Clinical - Whispered test in adults and children | Hearing impairment | ENT | Geriatrics |
| Price | 2005 | Biochem - Urine - Protein/Creatinine ratio | Renal - Proteinuria | Nephrology | Primary care |
| Puli | 2008^a^ | Radiology - Ultrasound - Endoscopic | Cancer - Gastric - N Stage | Gastroenterology | Oncology |
| Puli | 2008^b^ | Radiology - Ultrasound - Endoscopic | Mediastinal lymphadenopathy abnormalities | Gastroenterology | Oncology |
|  |  | Radiology - Ultrasound - Endoscopic - FNA | Mediastinal lymphadenopathy abnormalities | Gastroenterology | Oncology |
| Puli | 2008^c^ | Radiology - CT | Cancer - prostate cancer - stage of pelvic lymph nodes | Urology | Oncology |
|  |  | Radiology - MR | Cancer - prostate cancer - stage of pelvic lymph nodes | Urology | Oncology |
| Puli | 2008^d^ | Radiology - Ultrasound - Endoscopic | Cancer - Oesophageal - Metastasis | Gastroenterology | Oncology |
| Puli | 2007 | Radiology - Ultrasound - Endoscopic | Cancer - Vascular invasion in pancreatic and periampullary cancers | Gastroenterology | Oncology |
| Purkayastha | 2007^a^ | Radiology - CT - colonography | Cancer - Colorectal | Surgery | Gastroenterology |
|  |  | Radiology - MR - colonography | Cancer - Colorectal | Surgery | Gastroenterology |
| Purkayastha | 2006 | Biochem - Serum - Procalcitonin in patients with pancreatitis | Gastro/Surg - Pancreatitis - Acute - Severity | Surgery | Surgery |
| Purkayastha | 2005 | Radiology - MR - colonography | Cancer - Colorectal | Infectious diseases | Surgery |
| Purkayastha | 2007^b^ | Radiology - MR - Pre-operative assessment | Cancer - Rectal tumour - Size of circumferential margin | Surgery | Gastroenterology |
| Reese | 2006 | Biochem - Serum - ASCA | Gastro/Surg - Inflammatory bowel disease | Gastroenterology | Gastroenterology |
|  |  | Biochem - Serum - p-ANCA | Gastro/Surg - Inflammatory bowel disease | Gastroenterology | Gastroenterology |
| Roddam | 2005 | Biochem - Serum - cPSA | Cancer - Prostate | Urology | Primary care |
|  |  | Biochem - Serum - f/tPSA | Cancer - Prostate | Urology | Primary care |
|  |  | Biochem - Serum - PSA | Cancer - Prostate | Urology | Primary care |
| Rodgers | 2006 | Biochem - Urine - Bladder tumor marker (BTA) | Cancer - Bladder cancer | Primary care | Emergency medicine |
|  |  | Biochem - Urine - NMP22 | Cancer - Bladder cancer | Primary care | Emergency medicine |
|  |  | Cytology - urine | Cancer - Bladder cancer | General medicine | Urology |
|  |  | Bedside - Urine - Dipstick | Urology - Haematuria | Primary care | Emergency medicine |
|  |  | Radiology - CT | Urology - Haematuria - cause abnormality | General medicine | Urology |
|  |  | Micro - Phase contrast microscopy -urine | Urology - Haematuria - Glomerular disease | Primary care | Emergency medicine |
|  |  | Micro - Urine - Automated analysis - urine | Urology - Haematuria - Glomerular disease | General medicine | Urology |
|  |  | Microscopy - urine | Urology - Haematuria - Glomerular disease | General medicine | Urology |
|  |  | Bedside - Urine - Dipstick | Urology - Haematuria - Urinary calculi | General medicine | Urology |
| Ross | 2000 | Clinical - global impressions, | Sleep apnoea | Respiratory | Respiratory |
|  |  | clinical - prediction equns, | Sleep apnoea | Respiratory | Respiratory |
|  |  | Flow volume loops | Sleep apnoea | Respiratory | Respiratory |
|  |  | Oximetry | Sleep apnoea | Respiratory | Respiratory |
|  |  | Partial channel somnogram | Sleep apnoea | Respiratory | Respiratory |
| Roy | 2005 | Radiology - CT - Spiral | T/E - Pulmonary embolism | Cardiology | General medicine |
|  |  | Radiology - MR - Angiography | T/E - Pulmonary embolism | Cardiology | General medicine |
|  |  | Radiology - Ultrasound - Echocardiogram | T/E - Pulmonary embolism | Cardiology | General medicine |
|  |  | Radiology - Ultrasound - leg | T/E - Pulmonary embolism | Cardiology | General medicine |
|  |  | Radiology - VQ scan | T/E - Pulmonary embolism | Cardiology | General medicine |
| Safdar | 2005 | acridine orange leukocyte cytospin testing of IVD-drawn blood; | Infection - bacteraemia from IV line | General medicine | Surgery |
|  |  | Micro - Culture - qualitative catheter segment | Infection - bacteraemia from IV line | General medicine | Surgery |
|  |  | Micro - Culture - quantitative blood drawn through an IVD; | Infection - bacteraemia from IV line | General medicine | Surgery |
|  |  | Micro - Culture - quantitative catheter segment culture; | Infection - bacteraemia from IV line | General medicine | Surgery |
|  |  | Micro - Culture - semi-quantitative catheter segment (roll-plate method) | Infection - bacteraemia from IV line | Paediatrics | General medicine |
|  |  | Micro - Culture qualitative blood drawn through an IVD; | Infection - bacteraemia from IV line | Paediatrics | General medicine |
|  |  | Micro - Serum - Differential time to positivity of concomitant qualitative IVD-drawn and peripheral blood cultures ( 2 hours) | Infection - bacteraemia from IV line | Paediatrics | Surgery |
|  |  | Micro - Serum - paired quantitative peripheral and IVD-drawn blood cultures; | Infection - bacteraemia from IV line | Paediatrics | Surgery |
| Samson | 2002 | Radiology - FDG-PET - in women with abnormal mamm or physical exam | Cancer - Breast cancer | Surgery | Surgery |
| Sarmiento | 2003 | Micro - Bronchial aspirate - PCR | Infection - Tuberculosis - pulmonary - smear negative | Infectious diseases | Infectious diseases |
|  |  | Micro - Sputum - PCR | Infection - Tuberculosis - pulmonary - smear negative | Infectious diseases | Infectious diseases |
| Sauerland | 2004 | Clinical - examination in blunt trauma patients - Adults | Orthopaedic - Pelvic fractures | Emergency medicine | Orthopaedics |
|  |  | Clinical - examination in blunt trauma patients - children | Orthopaedic - Pelvic fractures | Emergency medicine | Orthopaedics |
| Scholten | 2001 | Clinical - Joint effusion test | Orthopaedic - Meniscal lesions of the knee | Orthopaedics | Orthopaedics |
|  |  | Clinical - joint line tenderness | Orthopaedic - Meniscal lesions of the knee | Orthopaedics | Orthopaedics |
|  |  | Clinical - McMurray | Orthopaedic - Meniscal lesions of the knee | Orthopaedics | Orthopaedics |
| Scholten | 2003 | Clinical - Anterior drawer test | Orthopaedic - Anterior cruciate ligament rupture | Orthopaedics | Orthopaedics |
|  |  | Clinical - Lachman tests | Orthopaedic - Anterior cruciate ligament rupture | Orthopaedics | Orthopaedics |
|  |  | Clinical - Pivot shift tests in GPs | Orthopaedic - Anterior cruciate ligament rupture | Orthopaedics | Orthopaedics |
| Schreiber | 2003 | Fluroscopy trans needle aspiration | Cancer - Lung cancer | Respiratory | Respiratory |
|  |  | Radiology - CT guided trans needle aspiration | Cancer - Lung cancer | Respiratory | Respiratory |
| Selman | 2005 | Radiology - CT | Cancer - Vulval - Lymph node metastasis | Oncology | Gynaecology |
|  |  | Radiology - MR | Cancer - Vulval - Lymph node metastasis | Oncology | Gynaecology |
|  |  | Radiology - PET | Cancer - Vulval - Lymph node metastasis | Oncology | Gynaecology |
|  |  | Radiology - Sentinal LN detction through blue dye & Tc-99 | Cancer - Vulval - Lymph node metastasis | Oncology | Gynaecology |
|  |  | Radiology - Ultrasound | Cancer - Vulval - Lymph node metastasis | Oncology | Gynaecology |
|  |  | Radiology - Ultrasound guided fine needle aspiration | Cancer - Vulval - Lymph node metastasis | Oncology | Gynaecology |
| Selman | 2008^a^ | Radiology - PET | IHD - Coronary artery disase | Cardiology | Cardiology |
| Selman | 2008^b^ | Radiology - CT | Cancer - Endometrial - lymph node status | Gynaecology | Oncology |
|  |  | Radiology - MR | Cancer - Endometrial - lymph node status | Gynaecology | Oncology |
|  |  | Sentinel node | Cancer - Endometrial - lymph node status | Gynaecology | Oncology |
| Shafiq | 2005 | Biochem - Serum procalcitonin | Gastro/Surg - Pancreatitis - Acute - Severity | Surgery | Surgery |
| Shaheen | 2008 | Biochem - Serum - AST-platelet ratio index (APRI) | Gastro/Surg - Liver fibrosis - Cirrhosis -Hepatitis C/HIV related | Hepatology | Infectious diseases |
|  |  | Biochem - Serum - AST-platelet ratio index (APRI) | Gastro/Surg - Liver fibrosis - significant - Heptatitis C/HIV related | Hepatology | Infectious diseases |
| Shaheen | 2007^a^ | Biochem - Serum - AST-platelet ratio index (APRI) | Gastro/Surg - Liver fibrosis - Hepatitis C related | Gastroenterology | Hepatology |
|  |  | Biochem - Serum - AST-platelet ratio index (APRI) | Gastro/Surg - Liver fibrosis - Hepatitis C related - Cirrhosis | Gastroenterology | Hepatology |
| Shaheen | 2007^b^ | Biochem - Serum - FibroTest (5 serum biochem markers) | Gastro/Surg - Liver fibrosis - Hepatitis C related - F2-F4 | Hepatology | Hepatology |
|  |  | Radiology - Ultrasound - FibroScan - transient elastography | Gastro/Surg - Liver fibrosis - Hepatitis C related - F2-F4 | Hepatology | Hepatology |
| Shi | 2008 | Biochem - Pleural fluid - CEA | Cancer - lung metastasis - Malignant pleural effusion | Respiratory | Oncology |
|  |  | Biochem - Pleural fluid - CEA | Cancer - mesothelioma - Malignant pleural effusion | Respiratory | Oncology |
| Shie | 2008 | Radiology - Bone scintography | Cancer - Breast cancer - Bone metastases | Oncology | Surgery |
|  |  | Radiology - FDG - PET | Cancer - Breast cancer - Bone metastases | Oncology | Surgery |
| Shiga | 2006 | Radiology - CT - helical | IHD - Thoracic aortic dissection | Cardiology | Cardiology |
|  |  | Radiology - MR | IHD - Thoracic aortic dissection | Cardiology | Cardiology |
|  |  | Radiology - Ultrasound - Echocardiogram - transoesophageal | IHD - Thoracic aortic dissection | Cardiology | Cardiology |
| Song | 2005 | Biochem - Serum - PSA | Cancer - Prostate cancer | Urology | Primary care |
|  |  | Clinical - per rectal examination | Cancer - Prostate cancer | Urology | Urology |
|  |  | Radiology - Ultrasound - trans-rectal | Cancer - Prostate cancer | Urology | Primary care |
| Sosna | 2008 | Radiology - CT - colonography | Gastro/Surg - Colorectal polyps >10mm | Gastroenterology | Surgery |
|  |  | Radiology - X-ray - Barium enema - Double contrast | Gastro/Surg - Colorectal polyps >10mm | Gastroenterology | Surgery |
| Sotiriadis | 2003 | Radiology - Ultrasound - Foetal - Intracardiac foci in foetuses | Obstetric - Down syndrome | Obstetrics | Obstetrics |
| Speight | 2006 | Clinical - examination by GP or GDP | Oral cancer and oral pre-cancer | Primary care | Primary care |
| St John | 2006 | Bedside - Urine - dipstick - Leucocyte esterase | Infection - Urinary tract | Obstetrics | General medicine |
|  |  | Bedside - Urine - dipstick - Leucocyte esterase AND nitrite | Infection - Urinary tract | Emergency medicine | Primary care |
|  |  | Bedside - Urine - dipstick - Leucocyte esterase OR nitrite | Infection - Urinary tract | Obstetrics | General medicine |
|  |  | Bedside - Urine - dipstick - Nitrite | Infection - Urinary tract | Emergency medicine | Primary care |
| Stein | 2006 | Radiology - CT - 16 slice | IHD - Coronary artery disase | Cardiology | Cardiology |
|  |  | Radiology - CT - 4 slice | IHD - Coronary artery disase | Cardiology | Cardiology |
|  |  | Radiology - CT - 64 slice | IHD - Coronary artery disase | Cardiology | Cardiology |
|  |  | Radiology - CT - 8 slice | IHD - Coronary artery disase | Cardiology | Cardiology |
| Stein | 2004 | Haem - Serum - D-dimer | T/E - Deep Venous Thrombosis | General medicine | General medicine |
|  |  | Haem - Serum - D-dimer | T/E - Pulmonary embolism | General medicine | General medicine |
| Steingart | 2007^a^ | Micro - Serum - antibody tests - Commercial | Infection - Tuberculosis - Extrapulmonary | Infectious diseases | Infectious diseases |
| Steingart | 2007^b^ | Micro - Serum - antibody test - commercial | Infection - Tuberculosis - pulmonary - smear negative | Respiratory | Infectious diseases |
|  |  | Micro - Serum - antibody test - commercial | Infection - Tuberculosis - pulmonary - smear positive | Respiratory | Infectious diseases |
| Steingart | 2006 | Micro - Sputum - Microscopy of smear with processing | Infection - Tuberculosis | Infectious diseases | Respiratory |
|  |  | Micro - Sputum - Microscopy of smear without processing | Infection - Tuberculosis | Infectious diseases | Respiratory |
| Stengel | 2005 | Radiology - Ultrasound - abdomen - Focussed assessment following trauma - adults | Gastro/Surg - Intraabdominal injury - organ | Surgery | Emergency medicine |
|  |  | Radiology - Ultrasound - abdomen - Focussed assessment following trauma - children | Gastro/Surg - Intraabdominal injury - organ | Surgery | Emergency medicine |
|  |  | Radiology - Ultrasound - abdomen - Focussed assessment following trauma - adults | Gastro/Surg - Intraperiotoneal fluid | Surgery | Emergency medicine |
|  |  | Radiology - Ultrasound - abdomen - Focussed assessment following trauma - children | Gastro/Surg - Intraperiotoneal fluid | Surgery | Emergency medicine |
| Stengel | 2001 | Radiology - Ultrasound - abdomen in blunt trauma in ED | Gastro/Surg - Intraabdominal injury - organ | Emergency medicine | Emergency medicine |
|  |  | Radiology - Ultrasound - abdomen in blunt trauma in ED | Gastro/Surg - Intraperiotoneal fluid | Emergency medicine | Emergency medicine |
| Takata | 2003 | Acoustic reflectometry | Infection - Otitis media with effusion in children | Paediatrics | ENT |
|  |  | Clinical - Otoscopy - pneumatic | Infection - Otitis media with effusion in children | Paediatrics | ENT |
|  |  | Clinical - Tympanometry - Portable | Infection - Otitis media with effusion in children | Paediatrics | ENT |
|  |  | Clinical - Tympanometry - Professional | Infection - Otitis media with effusion in children | Paediatrics | ENT |
| Tang | 2007 | Biochem - Serum - Procalcitonin | Infection - Sepsis | Critical care | Critical care |
| Terasawa | 2004 | Radiology - CT - abdomen - adults | Gastro/Surg - Appendicitis - acute | Surgery | Surgery |
|  |  | Radiology - CT - abdomen - teenagers | Gastro/Surg - Appendicitis - acute | Surgery | Surgery |
|  |  | Radiology - Ultrasound - abdomen - adults | Gastro/Surg - Appendicitis - acute | Surgery | Surgery |
|  |  | Radiology - Ultrasound - abdomen - teenagers | Gastro/Surg - Appendicitis - acute | Surgery | Surgery |
| Termaat | 2005 | Radiology - FDG - PET | Infection - Osteromyelitis - chronic (osteomyelitis requiring >1 episode of treatment and/or a persistent infection that had lasted > 6 wks | Orthopaedics | General medicine |
|  |  | Radiology - MR | Infection - Osteromyelitis - chronic (osteomyelitis requiring >1 episode of treatment and/or a persistent infection that had lasted > 6 wks | Orthopaedics | General medicine |
|  |  | Radiology - Scintography - Bone | Infection - Osteromyelitis - chronic (osteomyelitis requiring >1 episode of treatment and/or a persistent infection that had lasted > 6 wks | Orthopaedics | General medicine |
|  |  | Radiology - Scintography - Bone & Gallium | Infection - Osteromyelitis - chronic (osteomyelitis requiring >1 episode of treatment and/or a persistent infection that had lasted > 6 wks | Orthopaedics | General medicine |
|  |  | Radiology - Scintography - Bone & Leucocyte | Infection - Osteromyelitis - chronic (osteomyelitis requiring >1 episode of treatment and/or a persistent infection that had lasted > 6 wks | Orthopaedics | General medicine |
|  |  | Radiology - Scintography - Leucocyte | Infection - Osteromyelitis - chronic (osteomyelitis requiring >1 episode of treatment and/or a persistent infection that had lasted > 6 wks | Orthopaedics | General medicine |
|  |  | Radiology - X-ray | Infection - Osteromyelitis - chronic (osteomyelitis requiring >1 episode of treatment and/or a persistent infection that had lasted > 6 wks | Orthopaedics | General medicine |
| Tew | 2005 | Cytology - Intraoperative imprint cytology of sentinal node | Cancer - Breast - Lymph node metastasis | Surgery | Oncology |
| Trochez-Martinez | 2007 | Biochem - Serum - CRP in women with pre-term prelabour rupture of membranes | Infection -chorioamnioitis | Obstetrics | Obstetrics |
| Trowbridge | 2003 | Biochem - Serum - alkaline phosphatase | Gastro/Surg - Cholecystitis - Acute | Emergency medicine | Primary care |
|  |  | Biochem - Serum - Bilirubin | Gastro/Surg - Cholecystitis - Acute | Emergency medicine | Primary care |
|  |  | Biochem - Serum - Elevated ALT or AST | Gastro/Surg - Cholecystitis - Acute | Emergency medicine | Primary care |
|  |  | Clinical - Anorexia | Gastro/Surg - Cholecystitis - Acute | Emergency medicine | Primary care |
|  |  | Clinical - Emesis | Gastro/Surg - Cholecystitis - Acute | Emergency medicine | Primary care |
|  |  | Clinical - Fever (>35) | Gastro/Surg - Cholecystitis - Acute | Surgery | Gastroenterology |
|  |  | Clinical - Guarding | Gastro/Surg - Cholecystitis - Acute | Surgery | Gastroenterology |
|  |  | Clinical - Murphy sign | Gastro/Surg - Cholecystitis - Acute | Surgery | Gastroenterology |
|  |  | Clinical - Nausea | Gastro/Surg - Cholecystitis - Acute | Surgery | Gastroenterology |
|  |  | Clinical - Rebound | Gastro/Surg - Cholecystitis - Acute | Surgery | Gastroenterology |
|  |  | Clinical - Rectal tenderness | Gastro/Surg - Cholecystitis - Acute | Surgery | Emergency medicine |
|  |  | Clinical - Right upper quadrant mass | Gastro/Surg - Cholecystitis - Acute | Gastroenterology | Surgery |
|  |  | Clinical - Right upper quadrant pain | Gastro/Surg - Cholecystitis - Acute | Emergency medicine | Gastroenterology |
|  |  | Clinical - Right upper quadrant tenderness | Gastro/Surg - Cholecystitis - Acute | Emergency medicine | Gastroenterology |
|  |  | Haem - Serum - Leucocytosis | Gastro/Surg - Cholecystitis - Acute | Surgery | Emergency medicine |
|  |  | Haem - Serum - Leucocytosis and fever | Gastro/Surg - Cholecystitis - Acute | Surgery | Gastroenterology |
| Tse | 2008 | Radiology - Ultrasound - Endoscopic | Gastro/Surg - Gall stones in common bile duct | Gastroenterology | Surgery |
| Tuon | 2006 | Biochem - Pericardial fluid - Adenosine deaminase | Infection - Tuberculous pericarditis | Cardiology | Infectious diseases |
| Tuon | 2007 | Micro - Bronchial aspirate - PCR | Infection - Aspergillosis - Invasive | Respiratory | Infectious diseases |
| Vakil | 2006 | Clinical - Alarm features in general | Cancer - Gastrointenstinal - Upper - neoplasia | Primary care | Gastroenterology |
|  |  | Clinical - anaemia | Cancer - Gastrointenstinal - Upper - neoplasia | Primary care | Gastroenterology |
|  |  | Clinical - dysphagia | Cancer - Gastrointenstinal - Upper - neoplasia | Primary care | Gastroenterology |
|  |  | Clinical - Opinion | Cancer - Gastrointenstinal - Upper - neoplasia | Primary care | Gastroenterology |
|  |  | Clinical - weight loss | Cancer - Gastrointenstinal - Upper - neoplasia | Primary care | Gastroenterology |
| van Dongen | 2007 | Hysteroscopy in abnormal uterine bleeding | Gynaecol - Endometrial polyps - postmenopausal | Gynaecology | Gynaecology |
|  |  | Hysteroscopy in abnormal uterine bleeding | Gynaecol - Endometrial polyps - premenopausal | Gynaecology | Gynaecology |
|  |  | Hysteroscopy in abnormal uterine bleeding | Gynaecol - Uterine abnormalities | Gynaecology | Gynaecology |
|  |  | Hysteroscopy in abnormal uterine bleeding | Gynaecol -Sub-mucosal myomas | Gynaecology | Gynaecology |
| van Randen | 2008 | Radiology - CT | Gastro/Surg - Appendicitis - acute | Surgery | Surgery |
|  |  | Radiology - Ultrasound - Graded compression | Gastro/Surg - Appendicitis - acute | Surgery | Surgery |
| van Westreenen | 2004 | Radiology - FDG - PET | Cancer - Oesophageal - Metastasis (M) Stage | Gastroenterology | Oncology |
| van Zaane | 2008 | Radiology - Ultrasound - transoesophageal echocardiogram | IHD - atherosclerosis of ascending aorta | Cardiology | Cardiology |
| Vanezis | 2008 | ECG - Cornell criteria | IHD - Left ventricular hypertrophy | Cardiology | Primary care |
|  |  | ECG - Sokolow-Lyon criteria | IHD - Left ventricular hypertrophy | Cardiology | Primary care |
| Vanhoenacker | 2007 | Radiology - CT coronary angiography -Multi-detector | IHD - Coronary artery disease - Acute ACS & NSTEMI | Cardiology | Cardiology |
| Vasbinder | 2001 | Radiology - Captopril scintography | PVD - Renal artery stenosis | Nephrology | Nephrology |
|  |  | Radiology - CT angiography | PVD - Renal artery stenosis | Nephrology | Nephrology |
|  |  | Radiology - MR - Angiography - Gandolinium enhanced | PVD - Renal artery stenosis | Nephrology | Nephrology |
|  |  | Radiology - MR - Angiography - non enhanced | PVD - Renal artery stenosis | Nephrology | Nephrology |
|  |  | Radiology - Ultrasound | PVD - Renal artery stenosis | Nephrology | Nephrology |
|  |  | Therapeutic trial - Captopril test | PVD - Renal artery stenosis | Nephrology | Nephrology |
| Vestergaard | 2008 | Clinical - Dermoscopy | Cancer - melanoma | Dermatology | Dermatology |
|  |  | Clinical - Visual insepection | Cancer - melanoma | Dermatology | Dermatology |
| Virgili | 2007 | Radiology - Optical Coherence Tomography | Diabetic Macular oedema. | Ophthalmology | Ophthalmology |
| Vlaar | 2007 | Radiology - SPECT (Single photon emission CT) in suspected PD | Parkinsonian disease | Neurology | Neurology |
| von Roon | 2007 | Bicochem - Faecal - calprotectin | Cancer - Colorectal | Gastroenterology | Gastroenterology |
|  |  | Bicochem - Faecal - calprotectin | Gastro/Surg - Inflammatory Bowel Disease - Crohn's | Gastroenterology | Gastroenterology |
|  |  | Bicochem - Faecal - calprotectin | Gastro/Surg - Inflammatory Bowel Disease - Ulcerative Colitis | Gastroenterology | Gastroenterology |
| Vroomen | 1999 | Clinical - Crossed leg raise | Orthopaedic - Herniated discs - Sciatica | Orthopaedics | General medicine |
|  |  | Clinical - Lesegue test | Orthopaedic - Herniated discs - Sciatica | Orthopaedics | General medicine |
| Wang | 2008 | Micro - Urine - Antibody based detection test - ELISA | Infection - Helicobacter Pylori in children | Paediatrics | Paediatrics |
|  |  | Radiology - MR - Spectroscopy | Cancer - Prostate cancer | Urology | Urology |
| Wang | 2005 | Clinical - Therapeutic trial - Proton pump inhibitor | Gastro/Surg - GORD | Primary care | Gastroenterology |
| Wang | 2006 | Biochem - Serum - f/tPSA | Cancer - Prostate Cancer in Chinese | Urology | Urology |
|  |  | Biochem - Serum - tPSA | Cancer - Prostate Cancer in Chinese | Urology | Urology |
| Wardlaw | 2006 | Radiology - CT angiography | PVD - Carotid artery disease | Vascular surgery | Vascular surgery |
|  |  | Radiology - MR - Angiography | PVD - Carotid artery disease | Vascular surgery | Vascular surgery |
|  |  | Radiology - MR - Angiography - Contrast enhanced | PVD - Carotid artery disease | Vascular surgery | Vascular surgery |
|  |  | Radiology - Ultrasound | PVD - Carotid artery disease | Vascular surgery | Vascular surgery |
| White | 2000 | Radiology - CT angiography | PVD - Intracranial aneurysms | Neurosurgery | Neurology |
|  |  | Radiology - MR - Angiography | PVD - Intracranial aneurysms | Neurosurgery | Neurology |
|  |  | Radiology - Ultrasound - transcranial doppler | PVD - Intracranial aneurysms | Neurosurgery | Neurology |
| Whiting | 2006^a^ | Radiology - MR - brain | Multiple sclerosis | Neurology | Neurology |
| Whiting | 2006^b^ | Bedside - Urine - dipstick - Glucose | Infection - Urinary tract infection in children under 5 | Paediatrics | Primary care |
|  |  | Bedside - Urine - dipstick - Leucocyte esterase | Infection - Urinary tract infection in children under 5 | Paediatrics | Emergency medicine |
|  |  | Bedside - Urine - dipstick - Leucocyte esterase AND nitrite | Infection - Urinary tract infection in children under 5 | Paediatrics | Primary care |
|  |  | Bedside - Urine - dipstick - leucocyte esterase OR nitrite | Infection - Urinary tract infection in children under 5 | Paediatrics | Primary care |
|  |  | Bedside - Urine - dipstick - Nitrite | Infection - Urinary tract infection in children under 5 | Paediatrics | Emergency medicine |
|  |  | Micro - Urine - bacteriuria | Infection - Urinary tract infection in children under 5 | Paediatrics | Emergency medicine |
|  |  | Micro - Urine - Clean voided midstream sampling | Infection - Urinary tract infection in children under 5 | Paediatrics | Emergency medicine |
|  |  | Micro - Urine - culture | Infection - Urinary tract infection in children under 5 | Paediatrics | Emergency medicine |
|  |  | Micro - Urine - Pyuria | Infection - Urinary tract infection in children under 5 | Paediatrics | Primary care |
|  |  | Micro - Urine - pyuria AND bacteriuria | Infection - Urinary tract infection in children under 5 | Paediatrics | Primary care |
|  |  | Micro - Urine - pyuria OR bacteriuria | Infection - Urinary tract infection in children under 5 | Paediatrics | Emergency medicine |
| Whitsel | 2000 | ECG - QTc interval | Diabetes - Autonomic failure | Endocrinology | Endocrinology |
| Will | 2006 | Radiology - MR | Cancer - Lymph node metastases | Surgery | Oncology |
|  |  | Radiology - MR - nano-particle enhanced | Cancer - Lymph node metastases | Surgery | Oncology |
|  |  | Radiology - MR - nano-particle enhanced | Cancer - Lymph node metastases - Abdomen & pelvis | Surgery | Oncology |
|  |  | Radiology - MR - nano-particle enhanced | Cancer - Lymph node metastases - Chest | Surgery | Oncology |
|  |  | Radiology - MR - nano-particle enhanced | Cancer - Lymph node metastases - Head & Neck | Surgery | Oncology |
|  |  | Radiology - MR - nano-particle enhanced | Cancer - Lymph node metastases - Prostate | Surgery | Oncology |
| Williams | 2007 | Radiology - Ultrasound - Duplex | PVD - Renal artery stenosis | Nephrology | Nephrology |
| Wittkampf | 2007 | Clinical - PHQ-9 | Mental Health - Depression - Major depressive episode | Primary care | Psychiatry |
| Worster | 2008 | Biochem - Serum - NT-proBNP | IHD - Heart failure - symptomatic patients - acute dyspnoea | Emergency medicine | Emergency medicine |
| Worster | 2002 | Radiology - CT - helical | Renal stones - Acute | Urology | Urology |
|  |  | Radiology - Intravenous Pyelogram | Renal stones - Acute | Urology | Urology |
| Wykes | 2004 | Laparoscopy | Gynaecol - Endometriosis | Gynaecology | Gynaecology |

**Note**:

Tests and target disorders are given as broad category first (before dash), followed by detail of the test or target disorder.

Target disorders are categorised according to the ICD-10 classification

For patient settings the two most likely settings are given. These were based on evidence from the individual studies or where not available clinical experience.
